# Preventive role of cinnamaldehyde against tenuazonic acid- and Freund’s adjuvant-induced histopathological and biochemical alterations in the mouse model

**DOI:** 10.3389/fmicb.2023.1159881

**Published:** 2023-06-22

**Authors:** Ankita Kumari, Karuna Singh

**Affiliations:** Department of Zoology, MMV, Banaras Hindu University, Varanasi, India

**Keywords:** tenuazonic acid, Freund’s adjuvant, biochemical assays, histopathology, cinnamaldehyde, synergistic effect

## Abstract

**Introduction:**

This study was designed to assess the protective role of cinnamaldehyde (Cin) against the synergistic effect of tenuazonic acid (TeA) and Freund’s adjuvant on different organs of Swiss albino mice.

**Methods:**

TeA was administered singly and in combination with Freund’s adjuvant intra-peritoneally. The mice were divided into control (vehicle treated), mycotoxicosis-induced (MI) groups, and treatment groups. The route of administration of TeA was intra-peritoneal. The treatment group (FAICT) received Cin orally as a protective agent against TeA-induced mycotoxicosis. The effects on performance, differential leukocyte counts (DLC), and pathological measurements in eight organs (liver, lungs, kidney, spleen, stomach, heart, brain, and testis) were taken into consideration.

**Results:**

The body weight and feed consumption decreased significantly in the MI groups, which were reversed in the FAICT group. The necropsy observations revealed an increase in the relative organ-to-body weight percentage in the MI groups, which was restored to normal in the FAICT group. Freund’s adjuvant enhanced the effects of TeA on DLC. The antioxidant enzymes SOD and CAT decreased, while MDA increased in the MI groups. Caspase-3 activity was reduced in all organs and remained stable in the treatment group. TeA elevated the ALT concentration in the liver and kidneys and the AST in the liver, kidney, heart, and brain tissues. The oxidative stress induced by TeA in the MI groups was ameliorated in the treatment group. Histopathological observations consisted of NASH, pulmonary oedema and fibrosis, renal crystals and inflammation, splenic hyperplasia, gastric ulceration and cyst, cerebral axonopathy, testicular hyperplasia, and vacuolation in the MI groups. However, no such pathology was recorded in the treatment group.

**Discussions:**

Thus, it can be concluded that the toxicity of TeA was found to be enhanced when combined with Freund’s adjuvant. However, Cin exhibited promising protective effects against TeA + Freund’s adjuvant toxicity and reverted the pathological alterations caused by them. Additionally, this study emphasizes Freund’s adjuvant’s ability to increase mycotoxicity rather than just acting as an immunopotentiator.

## 1. Introduction

Mycotoxins are toxic secondary metabolites of microfungi that are ingested or inhaled by humans and other vertebrates (Alshannaq and Yu, 2017). Dermal toxicity and tumorigenesis are also caused due to dermal exposure of mycotoxins (Doi and Uetsuka, 2014). They are low molecular weight compounds that can cause acute and often chronic diseases when exposed to in small amounts for an extended period of time (Singh and Kumari, 2022).

Tenuazonic acid (TeA) is considered to have the highest toxicity among the *Alternaria* mycotoxins (Marchese et al., 2018). TeA is a product of tetramic acid and has been considered the etiological agent of Onyalai, a hemorrhagic condition that can cause severe bleeding into the mouth, palate, and intestinal mucosa (Gil-Serna et al., 2014).

Toxicity of TeA has been studied in several animals like mice, chicken, and dogs (Griffin and Chu, 1983; Kumari and Singh, 2021). At a concentration of 10 mg/kg body weight, it causes hemorrhages in dogs. Sub-acute toxicity was observed in chickens when fed at a dose of 10 μg/g of feed. In chickens, when the concentration of TeA was increased gradually from sub-lethal to lethal levels, it caused an increase in internal hemorrhage, a suppression in weight gain, and a reduction in feed efficiency (Griffin and Chu, 1983; Woody and Chu, 1992; Kumari and Tirkey, 2019). The oral LD_50_ for TeA in rats and mice lies in between 81 and 186 mg/kg (Gil-Serna et al., 2014). The mice, when fed with TeA at a concentration of 25 mg/kg body weight per day for a period of 10 months, precancerous changes in the esophageal mucosa were observed (Kumari and Tirkey, 2019). TeA inhibited the incorporation of amino acids into proteins by interacting with the peptidyltransferase in ribosomes, both *in vivo* in rats and *in vitro* in Ehrlich ascites tumor and rat liver cells (Kumari and Tirkey, 2019).

The use of botanicals and their phytochemicals has proved to be a promising detoxifier against mycotoxins (Makhuvele et al., 2020). The use of cinnamon is linked to a number of health advantages, including antibacterial action (Kawatra and Rajagopalan, 2015), antioxidant activity (Makhuvele et al., 2020), prevention of cancer cell proliferation (Shen et al., 2012), and glucose control in diabetes (Anderson et al., 2015). In addition to natural compounds, several bacteria have also been identified by researchers that possess the ability to degrade and/or adsorb OTA (Chen et al., 2018).

Cinnamaldehyde (Cin) makes up nearly 98% of the essential oil in the bark of cinnamon trees (Chemical Book, 2019) which has earlier been reported to be an effective inhibitor of the growth of a variety of yeasts, filamentous molds, and dermatophytes (Shreaz et al., 2016). The production of Ochratoxin A (OTA) was found to be decreased by Cin (Wang et al., 2018). Cin is also known to damage the membrane permeability of *A. alternata* and lower the concentration of alternariol (AOH) and alternariol monomethyl ether (AME; Xu et al., 2018). Cin can reduce the expression of the gene that produces aflatoxin (AF), thereby preventing the manufacture of AF B1 (Liang et al., 2015; Tian et al., 2018). Cin has also been recommended as a fungicide after the plantation of crops to control the production of mycotoxins (Awuchi et al., 2021). The antifungal activity of Cin has earlier been studied against *Aspergillus flavus* in both solid and liquid media. Cin was not only able to inhibit the growth of the molds but also the synthesis of AF B1 (Sun et al., 2016). Perczak et al. (2016) reported the degradation of ZEN with the use of a combination of essential oils, including cinnamon. Apart from being an anti-fungal agent, Cin has also been reported for its beneficial antioxidative and antiperoxidative effects. The protective role of Cin was demonstrated against the slow loss of pancreatic β-cells in diabetic rats (Subash-Babu et al., 2014).

Adjuvants are substances responsible for strong immunological responses (Centers for Disease Control and Prevention, 2022). The balance between adjuvant properties and adverse reactions plays an essential role in the choice of adjuvant for a study (Apostolico et al., 2016). Freund’s adjuvant is used as an immunopotentiator and is effective in stimulating cell-mediated immunity (Mohan et al., 2013).

In this study, the prophylactic effect of Cin against the toxicity of TeA + Freund’s adjuvant in various organs has been evaluated by assessing biochemical parameters such as superoxide dismutase (SOD), catalase (CAT), malondialdehyde (MDA), alanine aminotransferase (ALT), and aspartate aminotransferase (AST) enzymes. Cell apoptosis was assessed by measuring the Caspase-3 (Cas 3) enzyme.

## 2. Materials and methods

### 2.1. Animals

Twelve-week-old male Swiss mice (C3HHC strain), bred under pathogen-free conditions were used for the study. The study was performed in the Animal House Facility of the Department of Zoology (BHU, Varanasi, India). The use and care of the animals were in accordance with the guidelines and regulations set by the Institutional Animal Ethical Committee, Banaras Hindu University, India (BHU/DoZ/IAEC/2018-19/048).

### 2.2. Experimental design

**Figure 1:**
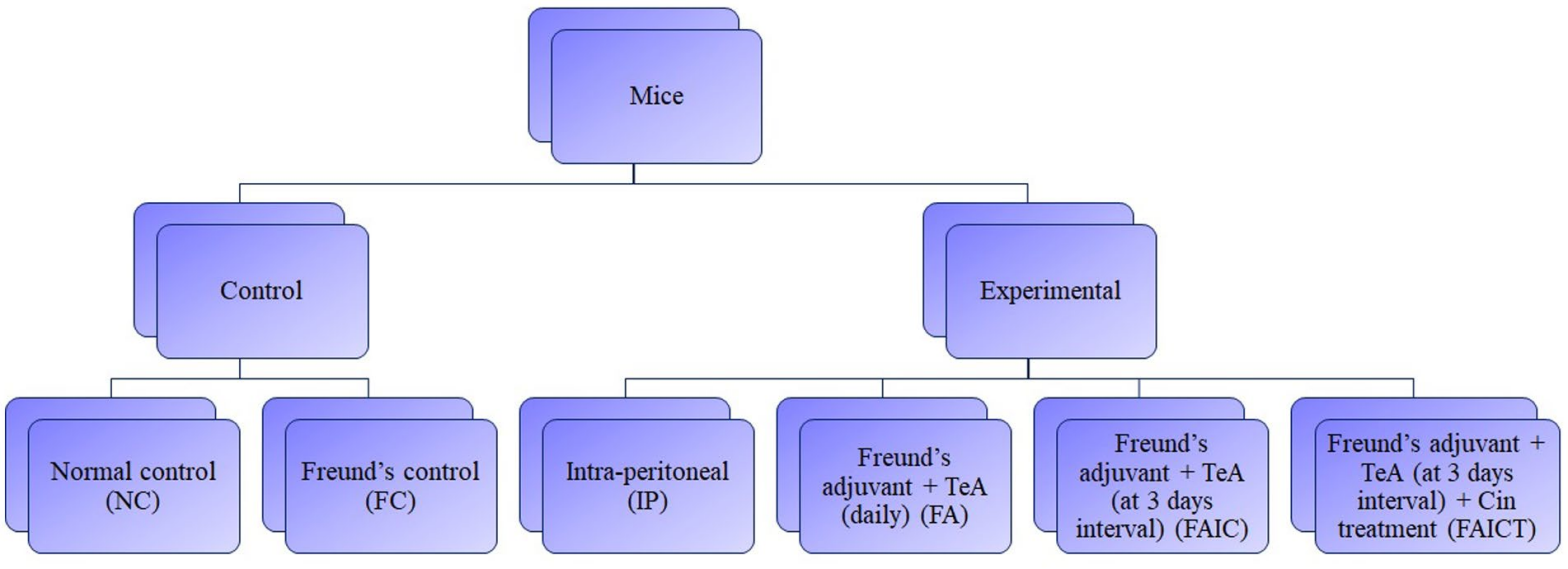
Flow-chart for grouping of mice. Dosing regimen-40 days, *n* = 5, Route-Intra-peritoneal injection (238 μg/kg BW/day), Treatment-Cinnamaldehyde (210 mg/kg BW/day). NC, Normal control mice, challenged with distilled water (intra-peritoneally) every day. FC, Freund’s control mice, challenged with distilled water in combination with complete Freund’s adjuvant and incomplete Freund’s adjuvant (intra-peritoneally) at intervals of 3  days. After one complete Freund’s adjuvant shot, two booster shots of incomplete Freund’s adjuvant were administered. IP, Intra-peritoneal TeA only. FA, TeA in combination with Freund’s adjuvant. Experimental mice, were challenged with TeA and Freund’s adjuvant through the intra-peritoneal route (IP) daily. After one complete Freund’s adjuvant shot, a booster shot of incomplete Freund’s adjuvant was administered on alternate days. FAIC, TeA in combination with Freund’s adjuvant. Experimental mice were challenged with TeA and Freund’s adjuvant through the intra-peritoneal route (IP) at intervals of 3  days. After one complete Freund’s adjuvant shot, two booster shots of incomplete Freund’s adjuvant were administered. FAICT, TeA in combination with Freund’s adjuvant. Experimental mice, challenged with TeA and Freund’s adjuvant through the intra-peritoneal route (IP) at intervals of 3  days. After 1 complete Freund’s adjuvant shot, 2 booster shots of incomplete Freund’s adjuvant were administered. At the 15th day, treatment with Cin (everyday) orally (PO) was started and was continued up to the 40th day.

To determine the potential toxicological effects, TeA (procured from Cayman Chemical, Item No. 11443) in combination with Freund’s adjuvant was injected to the mice. The study comprised of two control groups, three mycotoxicosis groups, and one treatment group. To study the TeA-related effects and Cin prophylaxis, five mice per group were allocated to each group-control, mycotoxicosis and treatment. The first control group (NC) received distilled water while the second control group (FC) received Complete Freund’s adjuvant (procured from Sigma-Aldrich F5881) and Incomplete Freund’s adjuvant (procured from Sigma-Aldrich F5506), without the mycotoxin on alternate days. The experimental mycotoxicosis group was further divided into three groups, one of which received only toxin (TeA), the second and the third received the mycotoxin in combination with Freund’s adjuvant. For the second mycotoxicosis-induced (MI) group (FA group), a 40-day repeated-dose TeA + Freund’s adjuvant study was conducted in which TeA was administered in combination with complete and TeA in combination with incomplete Freund’s adjuvants. In the third group [Freund’s adjuvant group (FAIC)], TeA was injected with complete (CFA) and incomplete Freund’s (IFA) adjuvants once every 3 days with alternate doses of CFA + TeA and IFA + TeA. The treatment group (FAICT) received the mycotoxin as administered to the FAIC group. In addition to TeA + Freund’s adjuvant, Cin (procured from HiMedia Laboratories GRM3277) was administered orally to the mice of the FAICT group starting at day 15 and continued up to day 40 (daily). The study was conducted in compliance with the Institutional Animal Ethics Committee (IAEC). Figure 1 shows a flowchart for the grouping of mice.

### 2.3. Determination of TeA dose

For *in vivo* toxicity studies, three doses of TeA, 119 μg/kg/day BW (low), 238 μg/kg BW (intermediate), and 476 μg/kg BW (high) were taken into consideration for the induction of sub-chronic TeA mycotoxicosis (Organisation for Economic Co-operation and Development, 2009; Kumari and Singh, 2021). Distilled water was used as a vehicle for TeA administration. A dose response survival-ship curve was plotted using Graphpad Prism 6 and the dosing regimen was carried out for 40 days.

### 2.4. TeA administration

TeA was isolated from *Paradendryphiella arenariae* (NCBI GenBank Accession no.-MW504999) cultures [Source—tomato (*Lycopersicon esculentum* Mill.)] as followed by Sanodiya et al. (2010) with minor modifications (Sanodiya et al., 2010). The methanolic extract of TeA was subjected to purification using thin layer chromatography (TLC) and high-pressure liquid chromatography (HPLC). The purity of the compound thus, obtained was checked using FT-IR and 1H-NMR. Further, ESI-MS and HRLC-MS were used to confirm the presence of TeA mycotoxin (Kumari and Singh, 2021; Kumari et al., unpublished data). A diagrammatic representation for the isolation and purification of TeA has been provided in Supplementary Figure 1. The route of administration in this study was intra-peritoneal injection. The mycotoxin (TeA) was typically mixed with an equal volume of the adjuvant to form an emulsion for administration with IFA and for administration with CFA, TeA was combined with CFA in the ratio of 3:1.

### 2.5. Determination of Cin dose

The dose determination for Cin treatment was carried out as previously described by Kumari and Singh (2021). Briefly, higher (420 mg/kg BW), intermediate (210 mg/kg BW), and lower (105 mg/kg BW) concentrations of Cin were administered for dose determination (Organisation for Economic Co-operation and Development, 2009). The stock solution of Cin was prepared at a concentration of 262.5 mg/mL of water (vehicle) and 20 μL of this was administered to mice. The mixture was vortexed before each administration to the mice and the administration of Cin was carried out for 56 days (Kumari and Singh, 2021). A Kaplan–Meier survival-ship curve for dose optimization was plotted using Graphpad Prism 6.

### 2.6. TeA mycotoxicosis induction with adjuvant and treatment

**Figure 2:**
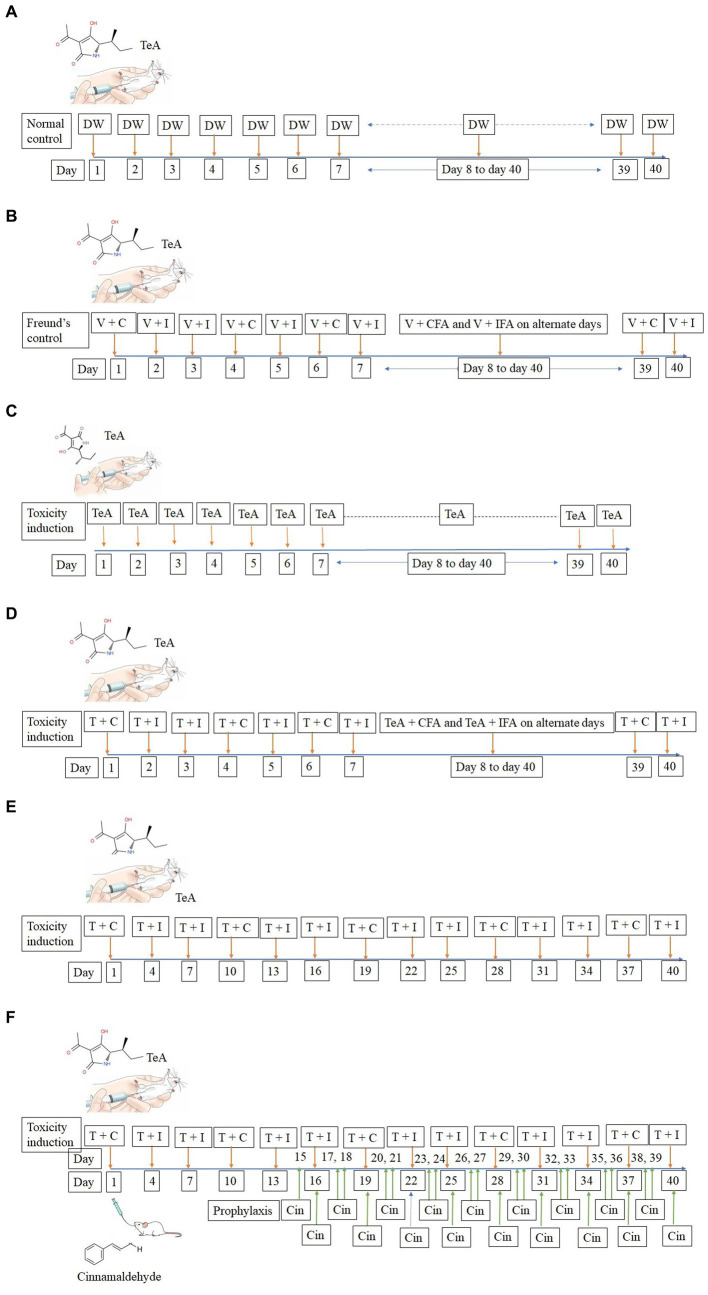
**(A)** Line diagram for dosing of mice in NC group; DW, Distilled water (vehicle). **(B)** Line diagram for dosing of mice in FC group; V + C − Distilled water (V − vehicle) + (C − Complete Freund’s Adjuvant), V + I − Distilled water (vehicle) + (I − Incomplete Freund’s Adjuvant). **(C)** Line diagram for dosing of mice in IP group; TeA. **(D)** Line diagram for dosing of mice in FA group; T + C − (T − TeA) + Complete Freund’s Adjuvant, T + I − TeA + Incomplete Freund’s Adjuvant. **(E)** Line diagram for dosing of mice in FAIC group, T + C − TeA + Complete Freund’s Adjuvant, T + I − TeA + Incomplete Freund’s Adjuvant. **(F)** Line diagram for dosing of mice in FAICT and Cin treatment group, T + C − TeA + Complete Freund’s Adjuvant, T + I − TeA + Incomplete Freund’s Adjuvant, Cin, Cinnamaldehyde treatment orally.

TeA was administered intra-peritoneally in mice at a concentration of 238 μg/kg BW. The normal control (NC) group received 50 μL DW intra-peritoneally, while the Freund’s control group (FC) received 50 μL injections of DW + CFA (3:1) and DW + IFA (1:1). The IP group received TeA at 238 μg/kg/day, the FA group received the same concentration (238 μg/kg/day) in combination with Freund′s adjuvants and the FAIC group received the dose in the interval of 3 days. The treatment group (FAICT) received Cin at a concentration of 210 mg/kg BW/day through oral administration, 2 weeks after the induction of mycotoxicosis (Kumari and Singh, 2021). The dosing regimen followed has been depicted below (Figure 2A—NC, Figure 2B—FC, Figure 2C—IP, Figure 2D—FA, Figure 2E—FAIC, Figure 2F—FAICT).

### 2.7. Biological parameters

The feed intake of mice was recorded every day along with their body weights. The enzymatic assays such as SOD (Das et al., 2000), CAT (Aebi, 1974), MDA (Ohkawa et al., 1979), AST (Reitman and Frankel, 1957), ALT (Reitman and Frankel, 1957), and Cas 3 (Srivastava et al., 2018) were determined. All the experimental mice were euthanized after 40 days of dosing (without diet deprivation). For differential leucocyte count (DLC), blood samples were collected by cardiac puncture. The weights of the organs were measured during dissection as absolute values, and the relative weights were then calculated according to body weights just before autopsy (Kumari and Singh, 2021).

$$Relativeorganweight=\frac{Organweight}{Bodyweight}*100\%$$

### 2.8. Histopathology

The organs (liver, lungs, kidney, spleen, stomach, heart, brain, and testis) were fixed in 10% neutral buffered formalin. The histological sections (6 μm) of the organs were cut and stained with hematoxylin and eosin (H&E). A Magnus DC 5 and Leica DM 2000 digital camera were used for photomicrography.

### 2.9. Statistical analyses and additional data analysis

The experimental groups were compared with the vehicle group and the Freund’s adjuvant control group whereas the treatment group FAICT was compared to the FAIC group (Sauder and DeMars, 2019; Kumari and Singh, 2021). Normally distributed data expressed as mean ± standard error mean (SEM) were compared using one-way analysis of variance (ANOVA) followed by post-hoc multiple comparison tests of Dunnett’s T3 or LSD for equal variances assumed or not assumed data, respectively. The difference was considered statistically significant at value of *p* less than 0.05 (Wang et al., 2022). These statistical analyses were performed using the IBM SPSS Statistics V25.0 software package.

## 3. Results

### 3.1. TeA dose optimization

**Figure 3:**
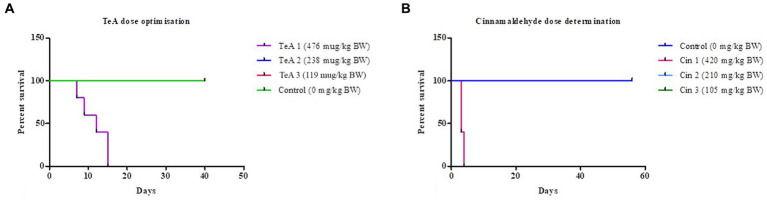
Survival-ship curve for **(A)** TeA dose administration at concentrations–Control (0 μg/kg BW), TeA 1 (476 μg/kg BW), TeA 2 (238 μg/kg BW), TeA 3 (119 μg/kg/day BW). **(B)** Cin dose determination at concentrations—Control (0 μg/kg BW), Cin 1 (420 mg/kg BW), Cin 2 (210 mg/kg BW), and Cin 3 (105 mg/kg BW).

The survival-ship curve in Figure 3A shows the dose dependent response of the mice where the mice of the control (distilled water, 0 μg/kg BW TeA) group was represented by green color, the mice of the TeA 1 (119 μg/kg BW) group in pink color (overlapped by green), TeA 2 (238 μg/kg BW) group in blue color (overlapped by green), and the TeA 3 (476 μg/kg BW) group in purple color. TeA at 238 μg/kg BW was found to be the effective dose to develop a sub-acute mycotoxicosis model. The mice died within 7 to 15 days of TeA injection at a concentration of 476 μg/kg BW while the lower dose (119 μg/kg BW) did not exhibit any adverse effect on the mice.

### 3.2. Cin dose optimization

The Kaplan–Meier curve for Cin dose determination has been shown in Figure 3B. The mice of the control group (distilled water, 0 mg/kg BW Cin) have been represented by a dark blue line. The administration of Cin at 210 mg/kg BW (Cin 2, light blue line, overlapped by dark blue line) provided best protection against TeA-induced mycotoxicity. However, the low dose (105 mg/kg BW; Cin 3, green line, overlapped by dark blue line) did not turn out to be effective while high dose (420 mg/kg BW; Cin 1, pink line) had deleterious effects on mice.

### 3.3. Behavioral and morphological observations

**Figure 4:**
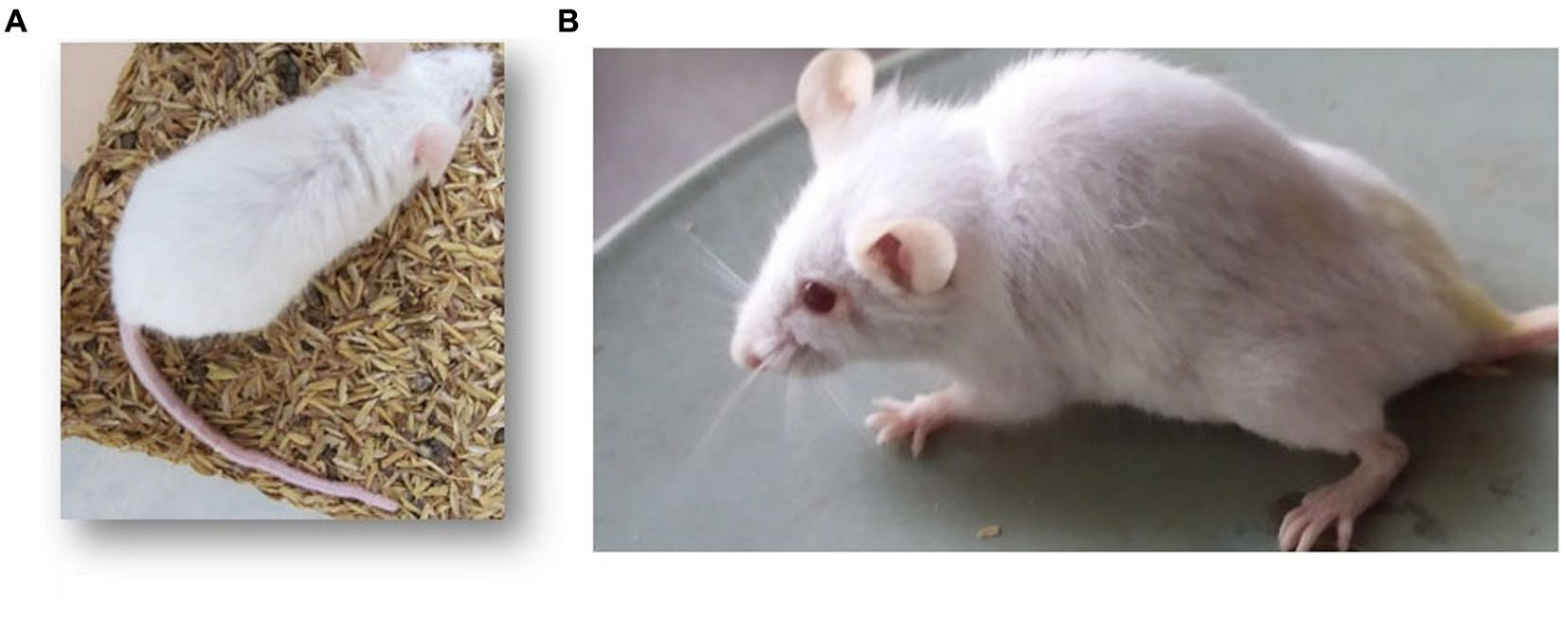
Mouse of the **(A)** control group with normal back, **(B)** FA group with kyphosis.

The mice of the FA, IP, and FAIC experimental groups exhibited lethargy. The mice of the FA group showed signs of kyphosis and hair loss as well (Figure 4).

### 3.4. Body weight and feed intake

**Figure 5:**
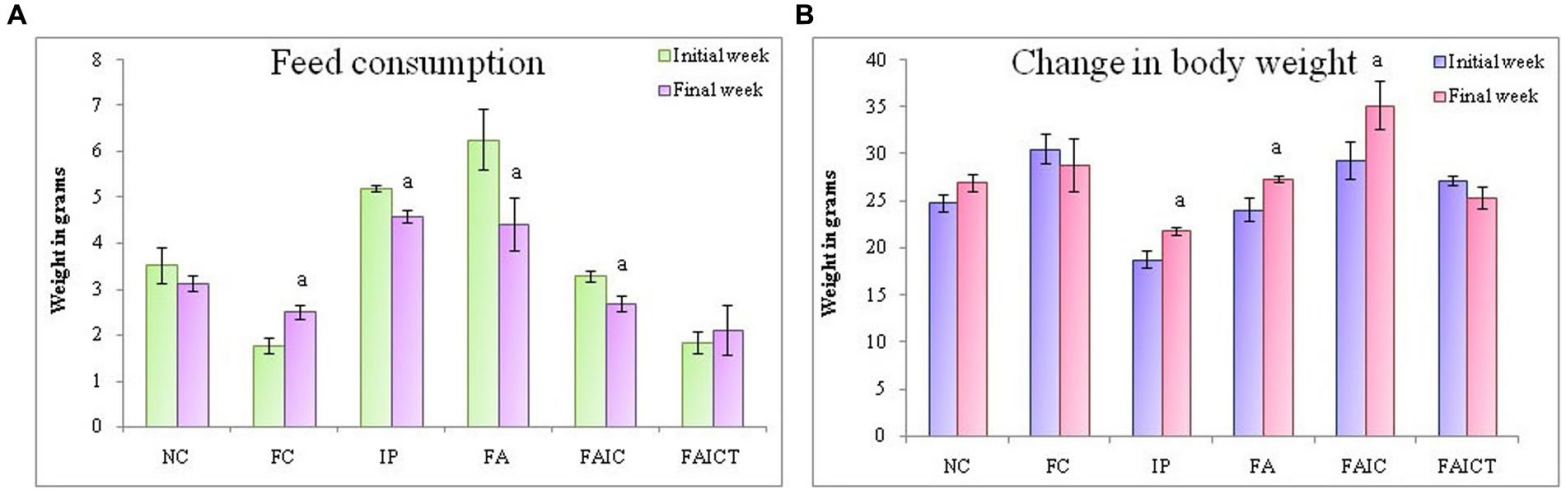
**(A)** Change in feed consumption in the initial and the final weeks; **(B)** Change in body weight in the initial and the final weeks. The values are mean ± SEM (*n* = 5); ^a^*p* ≤ 0.05 (statistically significant compared with initial week).

All the mycotoxicosis-induced groups (IP, FA, and FAIC) had decreased feed intake (Figure 5A). In Freund’s control group, feed consumption increased significantly. In comparison to the FAIC and IP groups, a notable decrease in feed intake was observed in the FA group. Similar to the normal control (NC) group, feed intake did not change after Cin treatment in the FAICT group (*p* ≤ 0.05, Figure 5A).

On comparing the BW of mice in the initial and final weeks of the experiment, a significant increase was noted in the IP, FA, and FAIC groups (Figure 5B). Both the control groups (NC and FC) and the treatment group (FAICT) did not show any change. Out of the three MI groups, FAIC showed the maximum increase in body weight, while the change in weight was almost equal in the FA and IP groups (Figure 5B). The difference in weight in the initial and final weeks was considered at 5% level of significance.

### 3.5. Differential leucocyte count

**Figure 6:**
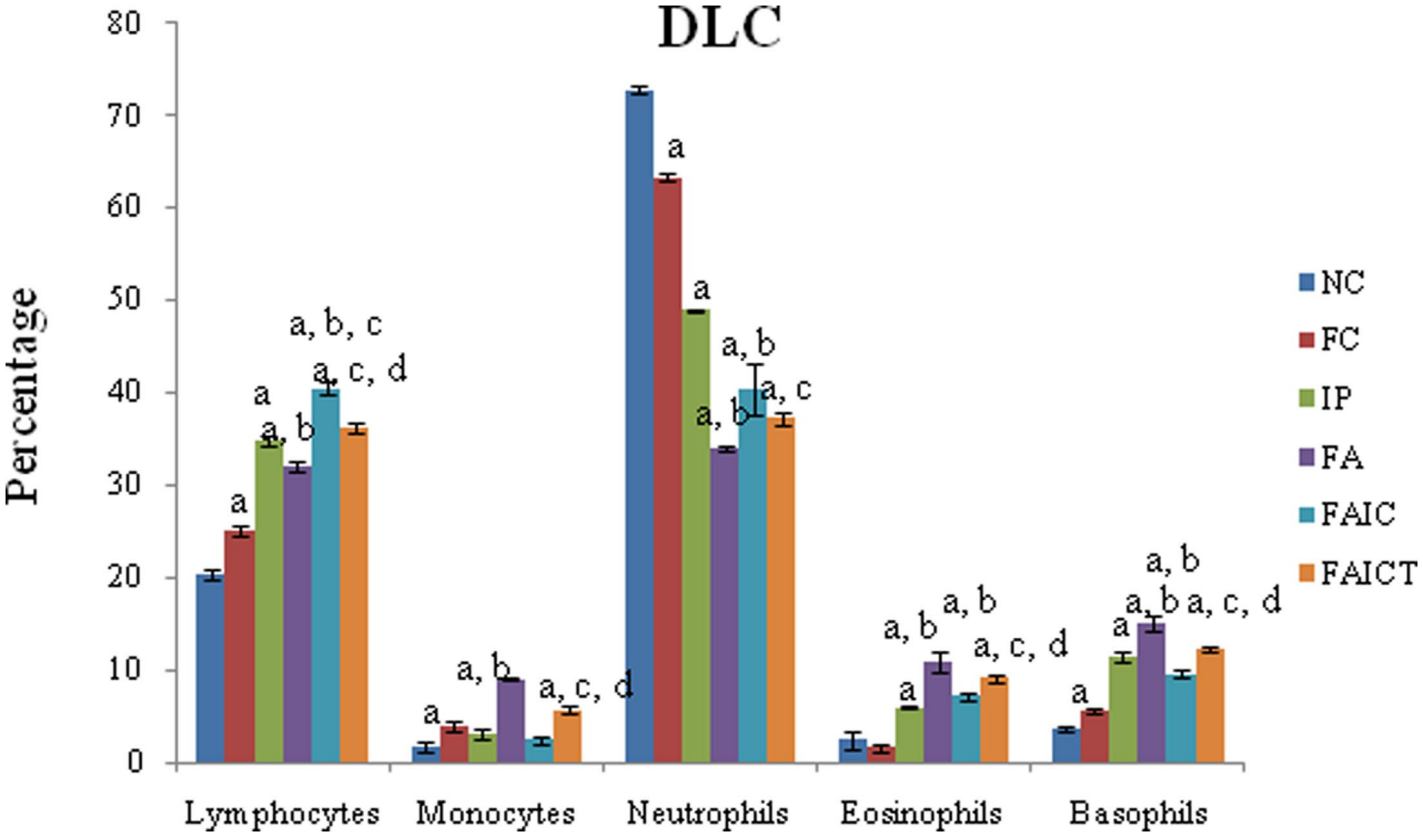
Comparison of differential leucocyte count in the control, MI and treated mice. The values are mean ± SEM where *n* = 5 [“a” symbolizes *p* ≤ 0.05 normal control vs. experimental groups, “b” symbolizes *p* ≤ 0.05 Freund’s control vs. experimental groups (FC vs FA, FAIC), “c” symbolizes *p* ≤ 0.05 MI vs. treatment groups (FA vs. FAICT), and “d” symbolizes *p* ≤ 0.05 MI vs. treatment groups (FAIC vs. FAICT)].

Although lymphocytosis and neutropenia were observed in all the MI groups (Figure 6), the DLC profile of the FA group exhibited an acute condition. Significantly elevated levels of monocytes, eosinophils, and basophils were also observed in all the MI groups (FA > IP > FAIC). The treatment of Cin decreased (*p* ≤ 0.05) the level of lymphocytes, monocytes, eosinophils, and basophils in the FAICT group, but could not bring it back to normal. On comparing the three MI groups, it was noted that the increase in lymphocyte count was more in IP and FAIC group as compared to the FA group (*p* ≤ 0.05). The percentage of lymphocyte was significantly more in FAIC in comparison to the IP group. While in case of neutrophils, the decrease (*p* ≤ 0.05) in neutrophil percentage was in the order FA < FAIC<IP.

### 3.6. Relative organ: body weight percentage

**Figure 7:**
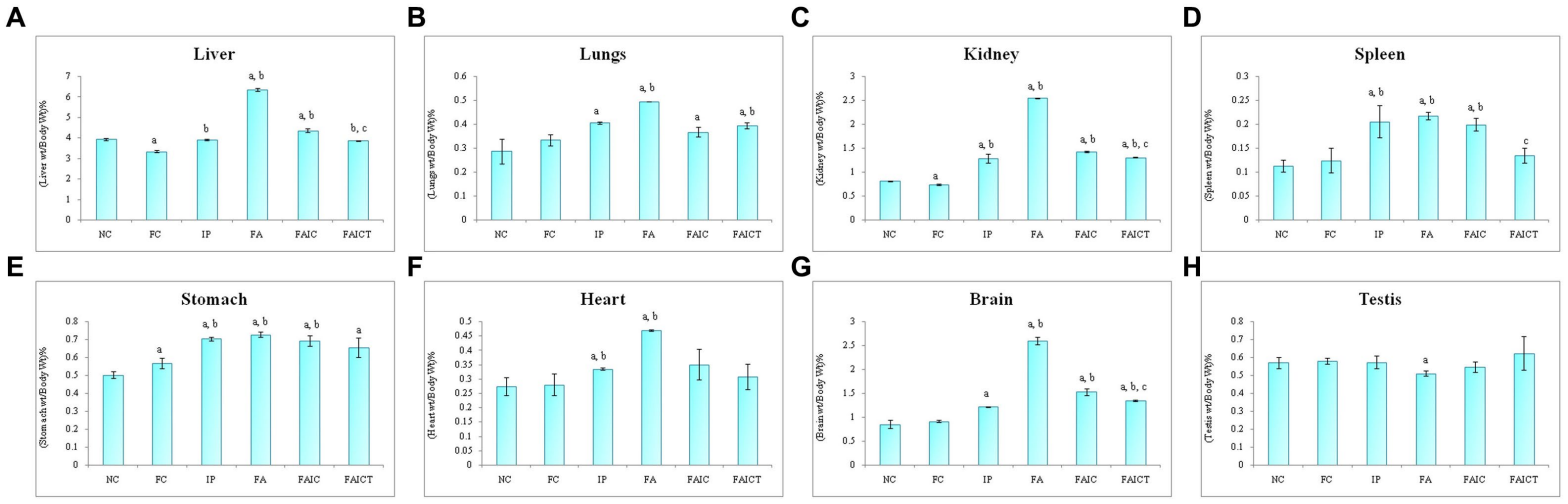
Relative organ: body weight percentage of **(A)** Liver, **(B)** Lungs, **(C)** Kidney, **(D)** Spleen, **(E)** Stomach, **(F)** Heart, **(G)** Brain, **(H)** Testis. Values are mean ± SEM where *n* = 5 [“a” symbolizes *p* ≤ 0.05 normal control vs. experimental groups, “b” symbolizes *p* ≤ 0.005 Freund’s control vs. experimental groups, “c” symbolizes *p* ≤ 0.05 MI vs. treatment groups (FAIC vs. FAICT)].

The weight of liver increased in the FA group, followed by the FAIC and IP groups (Figure 7A). The treatment of Cin, however, decreased the weight of the liver when compared to FA and FAIC group. The FC group had lower liver weight than the NC group (*p* ≤ 0.05).

The weight of the lungs was elevated in the MI groups in the order of FA > IP > FAIC (Figure 7B). The lungs of the FAICT group weighed lower in comparison to the FA group. No significant difference was noted between the NC and FC groups (*p* ≤ 0.05).

The kidneys also showed an increment in the weight of the MI groups in FA > FAIC > IP order (Figure 7C). The weight of the kidneys of the FAICT group was at par with the IP group but was low compared to the other two MI groups. A slight decrease in the weight of kidneys was noted in FC with respect to NC (*p* ≤ 0.05).

A significant increase in the weight of the spleen of all the MI groups was noted (Figure 7D), which was brought down in the treated group. No change in the splenic weight was recorded in the control groups (*p* ≤ 0.05).

All the three MI groups showed elevation in the weight of the stomach, which could not be brought down in the Cin treated group (Figure 7E). A slight increase in the stomach weight was recorded in the FC group as well (*p* ≤ 0.05).

An increase in the weight of the heart was observed in the FA and IP groups (FA > IP; Figure 7F). No significant change was recorded in any other group (*p* ≤ 0.05).

The increase in the relative weight of the brain was in FA > FAIC > IP order (Figure 7G). The relative weight of the brain decreased in the FAICT group when compared to the FA and FAIC groups. However, no change in the weight of the brain was observed in the FAICT group in comparison to the IP group. The FAICT group showed an increase in the relative weight of the brain when compared to the NC and FC groups. Both the control groups did not show any significant difference when compared to each other (*p* ≤ 0.05).

The weight of the testis of the FA group was found to be slightly lower in comparison to other groups (Figure 7H). No change in the weights of the testes was noticed in other groups (*p* ≤ 0.05).

### 3.7. Anatomical, histopathological and biochemical changes

#### 3.7.1. Liver

##### 3.7.1.1. Anatomical changes in the liver

**Figure 8:**
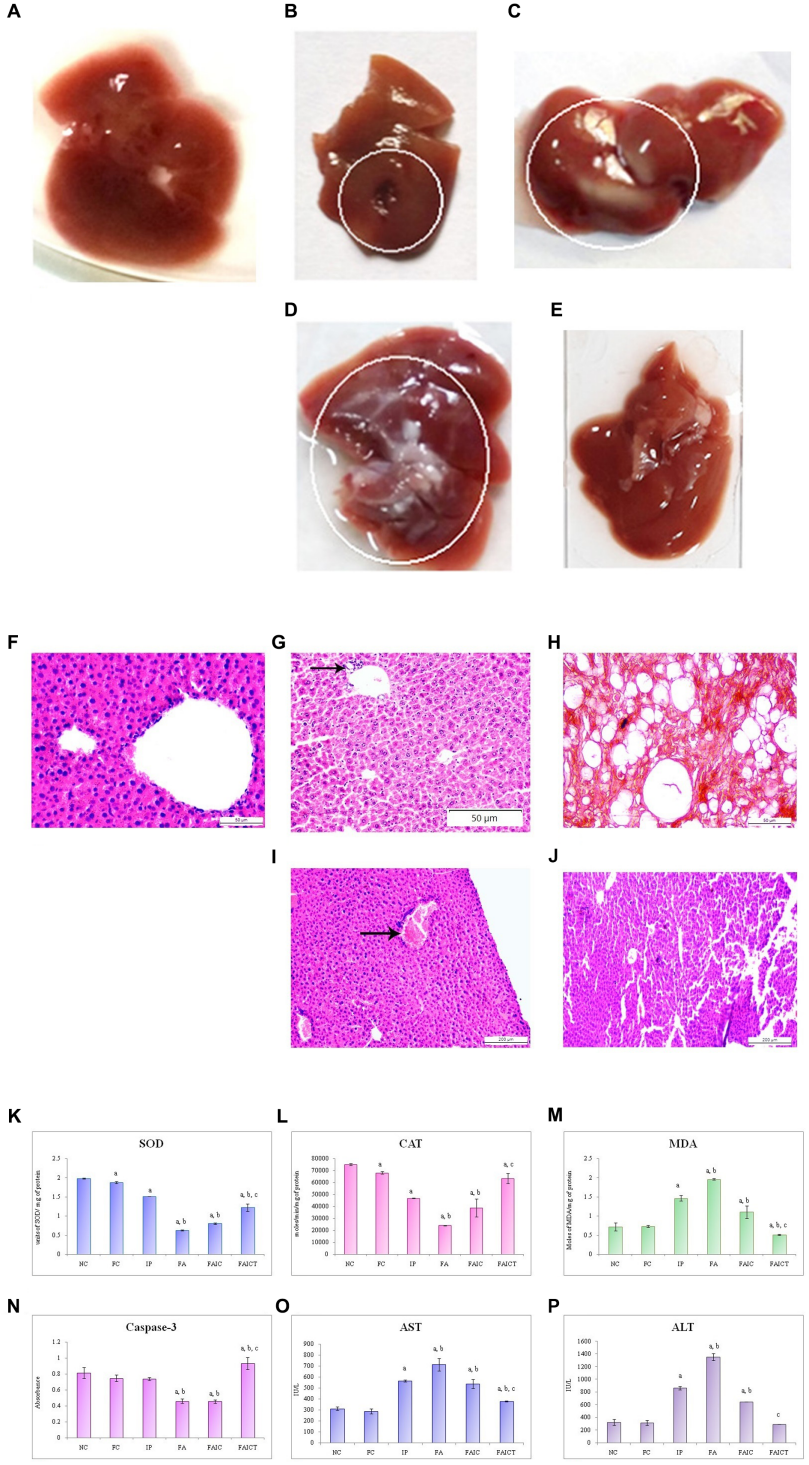
Liver of the **(A)** control, **(B)** IP group shows gross lesions, as indicated by the circle, **(C)** FA group with fatty depositions in liver as shown by a circle, and **(D)** FAIC group has some fatty depositions (encircled) **(E)** FAICT group with no pathology. T. S. liver of the **(F)** control mouse (HEx400) showing no pathology, **(G)** MI mouse-IP (HEx200) consisting of focal inflammation (arrow), **(H)** MI mouse-FA (HEx400) showing steatosis and ballooning, **(I)** MI mouse-FAIC (HEx100) showing inflammatory cells (arrow), and **(J)** treated mouse-FAICT (HEx100) showing normal histology. Comparison of **(K)** SOD activity in the liver of the control, MI and treated mice, **(L)** CAT activity in the liver of control, MI and treated mice, **(M)** MDA activity in the liver of control, MI and treated mice, **(N)** Caspase-3 activity in the liver of control, MI and treated mice, **(O)** AST activity in the liver of control, MI and treated mice, and **(P)** ALT activity in the liver of control, MI and treated mice. The results of biochemical assays are shown in mean ± SEM, *n* = 5, (“a” symbolizes *p* ≤ 0.005 normal control vs. experimental groups, “b” symbolizes *p* ≤ 0.05 Freund’s control vs. experimental groups, and “c” symbolizes *p* ≤ 0.05 MI vs. treatment group FAIC vs. FAICT).

The IP group showed the presence of gross lesions as shown in Figure 8B. Unlike the control group (Figure 8A), the anatomical changes in the liver after co-administration of Freund’s adjuvant and TeA mycotoxin revealed fatty changes and hepatic steatosis in the FA group (Figure 8C). The FAIC group showed mild fatty deposition on the tissue (Figure 8D). No such changes were observed in the treatment group (FAICT; Figure 8E).

##### 3.7.1.2. Histopathological changes in the liver

The control liver has been shown in Figure 8F. The histopathology of the liver of the IP group (Figure 8G) revealed focal inflammation. The FA group (Figure 8H) exhibited the presence of diffused fat globules mostly near the periphery of the hepatic tissue. Non-alcoholic fatty liver or steatosis and ballooning were also noted in the FA group. The FAIC group, however (Figure 8I) displayed the presence of the inflammatory cells. The treatment group (FAICT; Figure 8J) did not exhibit any pathology.

##### 3.7.1.3. Biochemical changes in the liver

SOD: The decreased SOD levels were noted in the liver of all the MI groups. The lowest level of SOD was observed in the FA group. The SOD in the FAIC group was slightly higher than in FA but was found to be lower than the IP group. The FAICT group had an increased SOD level as compared to FA and FAIC but was lower than the IP group. On comparing NC and FC, FC showed a decrease in SOD (Figure 8K; *p* ≤ 0.05).

CAT: CAT level declined significantly in the hepatic tissues of the MI groups (FA < FAIC ≤ IP). Cin treatment elevated its level but not equipollent to NC. The FC group had a lower CAT activity than NC (Figure 8L; *p* ≤ 0.05).

MDA: Lipid peroxidation (LPO) in liver was found to be higher in the MI groups in comparison to the control groups. FA showed the highest lipid peroxidase activity, followed by IP and then FAIC groups. The treatment group exhibited lowered lipid peroxidation activity. The FC group showed no significant difference as compared to the NC group (Figure 8M; *p* ≤ 0.05).

Cas 3: The hepatic tissues showed decreased Cas 3 activity in the FA and FAIC groups. No significant difference was noted between the FA and the FAIC group, while FAICT had an elevated level of Cas 3 in comparison to all other groups. The Freund’s control and IP groups did not show any significant change (Figure 8N; *p* ≤ 0.05).

AST and ALT: The increased levels of AST (Figure 8O) and ALT (Figure 8P) were recorded in the hepatic tissue in the MI groups (FA > IP > FAIC). The treatment with Cin significantly lowered the ALT and AST levels. No change was noted in the FC group (*p* ≤ 0.05).

#### 3.7.2. Lungs

##### 3.7.2.1. Anatomical changes in the lungs

**Figure 9:**
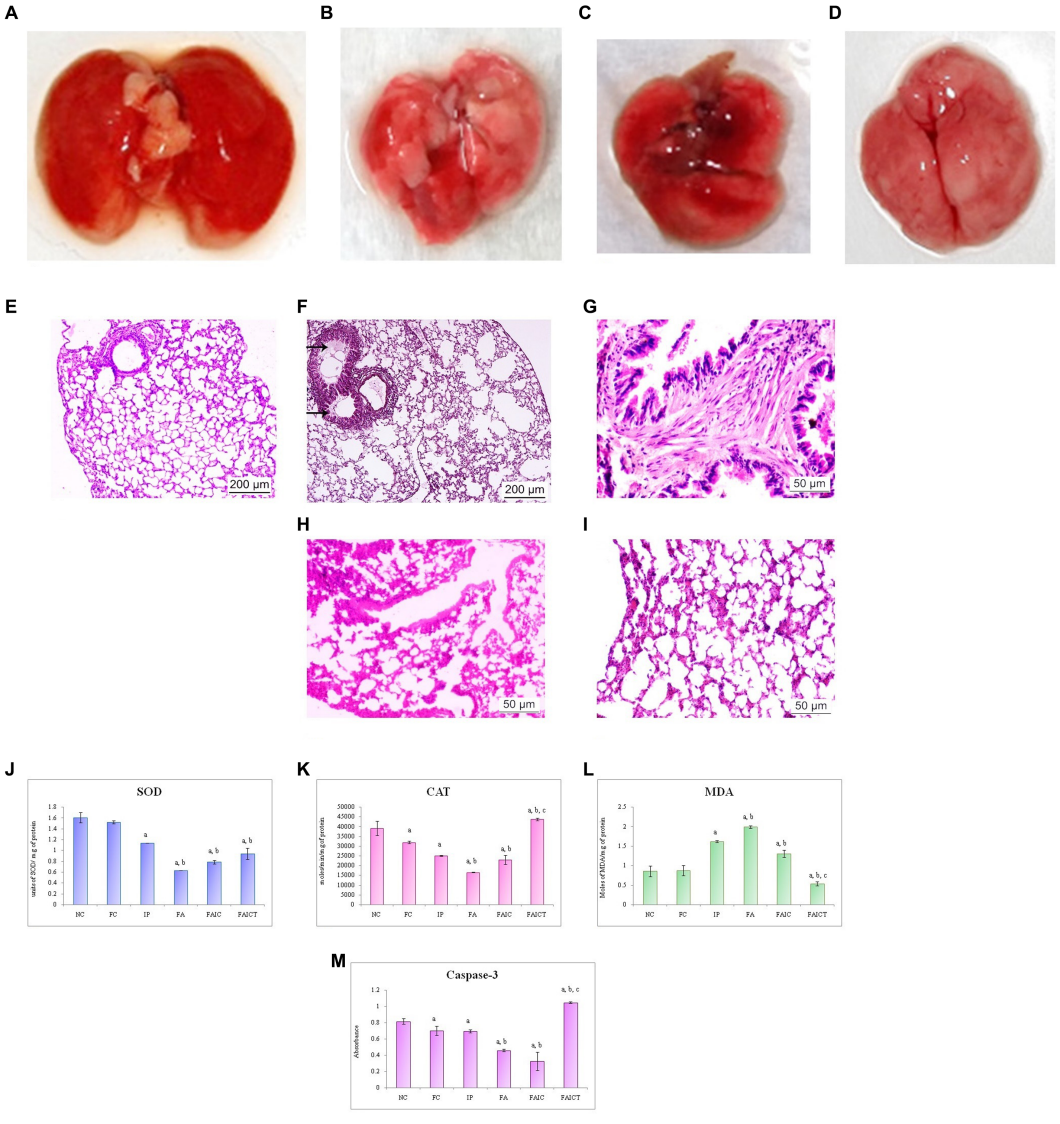
Lungs of the **(A)** control mouse, **(B)** IP group showing discoloration, **(C)** FA group showing lesions, and **(D)** FAIC group showing discoloration. T. S. lungs of the **(E)** control mouse (HEx100) showing no pathological changes, **(F)** MI mouse-IP (HEx100) showing oedema (arrows), **(G)** MI mouse-FA group (HEx400) showing fibrosis, **(H)** MI mouse-FAIC (HEx400) showing alveolar wall thickening, and **(I)** treatment mouse-FAICT (HEx400) showing normal architecture of the alveolar sacs. Comparison of **(J)** SOD activity in the lungs of the control, MI and treated mice, **(K)** CAT activity in the lungs of control, MI and treated mice, **(L)** MDA activity in the lungs of control, MI and treated mice, **(M)** Caspase-3 activity in the lungs of control, MI and treated mice. The results of biochemical assays are shown in mean ± SEM, *n* = 5 (“a” symbolizes *p* ≤ 0.05 normal control vs. experimental groups, “b” symbolizes *p* ≤ 0.05 Freund’s control vs. experimental groups and “c” symbolizes *p* ≤ 0.05 MI vs. treatment group FAIC vs. FAICT).

The lungs of the MI groups (IP and FAIC) showed discoloration (Figures 9B,D) when compared to that of the healthy control (Figure 9A). The gross lesions were observed on lungs of the FA group (Figure 9C).

##### 3.7.2.2. Histopathological changes in the lungs

The histopathology of the lungs revealed changes in all the MI groups. The control lungs had normal histology (Figure 9E), while the IP group showed the presence of oedema (Figure 9F). The FA (Figure 9G) group exhibited fibrosis in the lungs, while FAIC (Figure 9H) showed thickening of the alveolar walls. The FAICT group had normal architecture of the lung tissue (Figure 9I).

##### 3.7.2.3. Biochemical changes in the lungs

SOD: The lung tissues showed decreased SOD levels in all the MI groups in FA < FAIC < IP order. The treatment group showed more SOD activity than the FA group but remained equivalent to the IP and FAIC group. No significant difference was found among the control groups (FC vs. NC; Figure 9J; *p* ≤ 0.05).

CAT: The lungs also showed a significant lowering in the CAT enzyme in the MI groups, with FA being the most affected group. The treatment with Cin increased the level of CAT enzyme. Among the control group, CAT concentration in FC was less than that in NC (Figure 9K; *p* ≤ 0.05).

MDA: MDA levels in lungs were high in IP, FA, and FAIC groups. Cin treatment brought down MDA levels and the concentration of MDA was lowest in the treatment group. FC in comparison to NC did not show any change in MDA concentration (Figure 9L; *p* ≤ 0.05).

Cas 3: The FA, IP and FAIC groups exhibited a decrease in the Cas3 enzyme in the lung tissues. Its lowest concentration was recorded in FAIC. An elevated Cas 3 enzyme concentration was observed in the FAICT group. The FC group had a lower concentration of Cas 3 than NC. No significant change was noted in the IP group in comparison to the FC group (Figure 9M; *p* ≤ 0.05).

#### 3.7.3. Kidneys

##### 3.7.3.1. Histopathological changes in the kidneys

**Figure 10:**
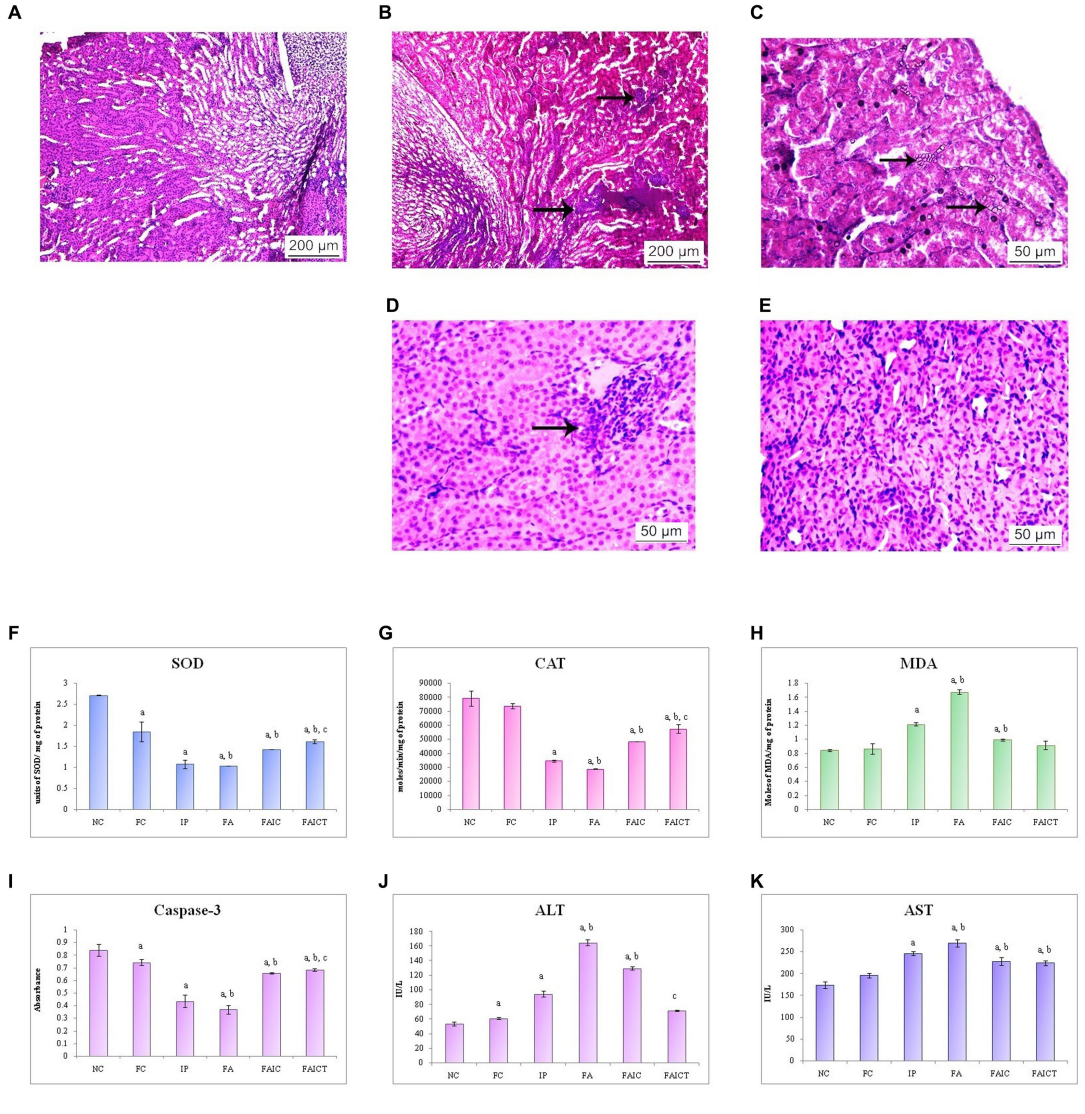
T. S. kidney of the **(A)** control mouse (HEx400) showing normal histology, **(B)** MI mouse-IP (HEx400) showing cellular infiltration (arrows), **(C)** MI mouse-FA (HEx400) showing presence of crystals in the renal tubules (arrows), **(D)** MI mouse-FAIC (HEx400) showing inflammation (arrow), and **(E)** treated mouse-FAICT (HEx400) showing no pathological changes. Comparison of **(F)** SOD activity in the kidney of the control, MI and treated mice, **(G)** CAT activity in the kidney of control, MI and treated mice, **(H)** MDA activity in the kidney of control, MI and treated mice, **(I)** Caspase-3 activity in the kidney of control, MI and treated mice, **(J)** ALT activity in the kidney of control, MI and treated mice, and **(K)** AST activity in the kidney of control, MI and treated mice. The results of biochemical assays are shown in mean ± SEM, *n* = 5 (“a” symbolizes *p* ≤ 0.05 normal control vs. experimental groups, “b” symbolizes *p* ≤ 0.05 Freund’s control vs. experimental groups and “c” symbolizes *p* ≤ 0.05 MI vs. treatment group FAIC vs. FAICT).

The co-administration of Freund’s adjuvant and TeA mycotoxin resulted in histopathological changes in the kidney as well. Cellular infiltration in the interstitium was observed in the kidney of the IP group (Figure 10B). The FA (Figure 10C) group revealed the presence of crystals in the cortical region, while the FAIC group (Figure 10D) showed inflammation. The kidneys of the control (Figure 10A) as well as FAICT groups (Figure 10E) exhibited normal histology.

##### 3.7.3.2. Biochemical changes in the kidney

SOD: The order of SOD concentration in kidneys was FA ≤ IP < FAIC. The kidneys showed a significantly higher level of SOD in the treatment group in comparison to the mycotoxicosis-induced groups. Further, the SOD level dropped in the FC group when compared to the NC group (Figure 10F; *p* ≤ 0.05).

CAT: The concentration of CAT in kidneys was reduced in the MI groups and was in FA < IP < FAIC order. A slight increase in the concentration of CAT was observed in the FAICT group in comparison to the MI groups. There was no significant difference between the levels of CAT in the control groups (NC vs. FC; Figure 10G; *p* ≤ 0.05).

MDA: The renal tissue showed high levels of MDA in the order FA > IP > FAIC. The treatment with Cin lowered the MDA level significantly in comparison to the MI groups. There was no significant difference between the two controls (Figure 10H; *p* ≤ 0.05).

Cas 3: Among the MI groups, the Cas 3 activity was in the order FA ≤ IP < FAIC in the kidneys. The concentration of Cas 3 was higher in FAICT with respect to the MI groups. The FC group, however, had a low concentration of Cas 3 when compared to NC group (Figure 10I; *p* ≤ 0.05).

AST and ALT: An increase in both ALT (Figure 10J) and AST (Figure 10K) enzymes was recorded in the renal tissues. The order of increase of ALT was FA > FAIC > IP while that for AST was FA > IP > FAIC. The treatment of Cin lowered the ALT enzyme as depicted in the FAICT group. However, in case of AST, no significant change was noted in the FAIC and FAICT group. FC group showed slightly higher ALT and almost similar level of AST enzyme in comparison to the NC (*p* ≤ 0.05).

#### 3.7.4. Spleen

##### 3.7.4.1. Histopathological changes in the spleen

**Figure 11:**
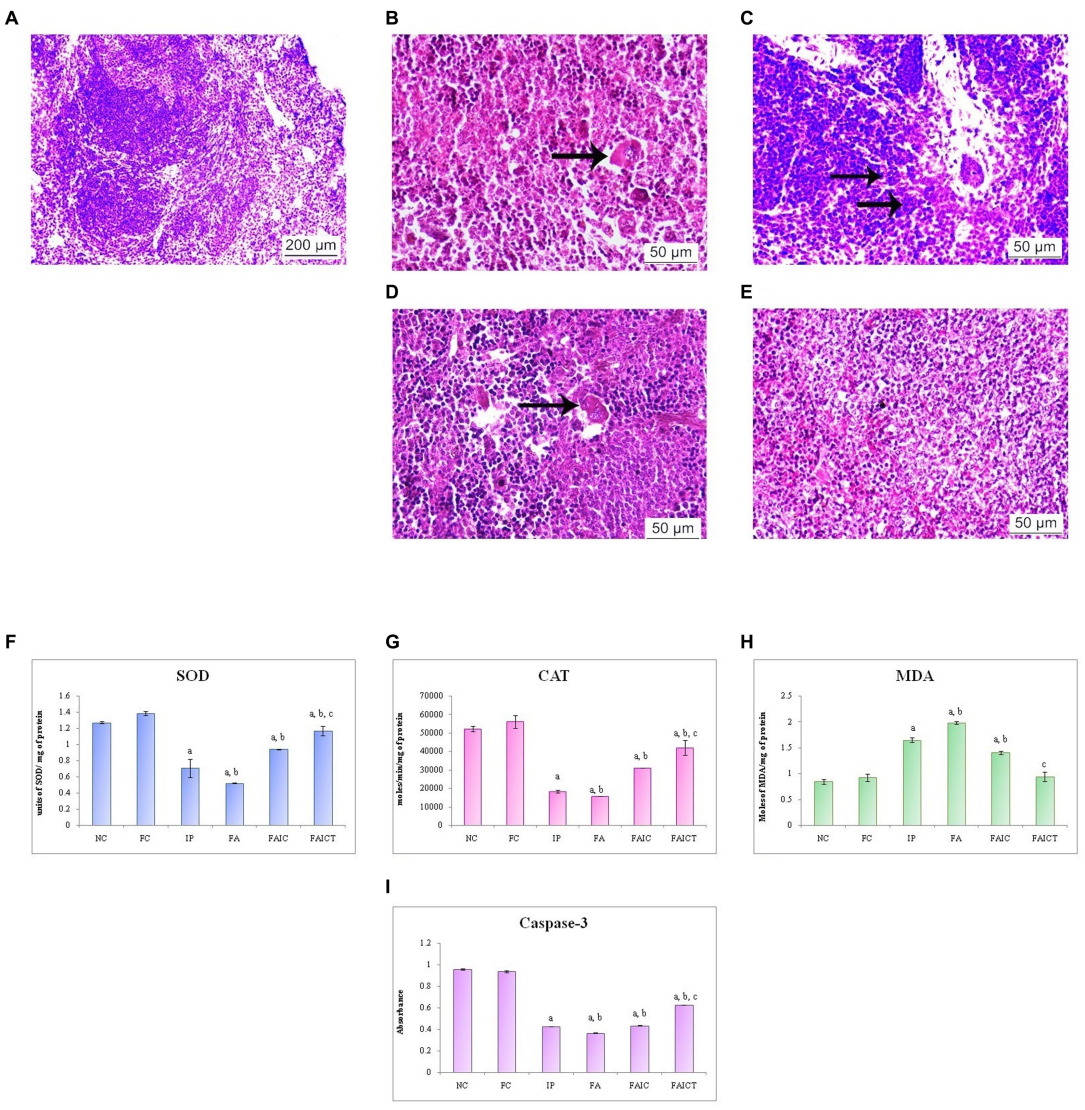
T. S. spleen of the **(A)** control mouse (HEx100) showing normal histology of red pulp and white pulp regions, **(B)** IP group (HEx400) showing erythrophagocytosis (arrow), **(C)** FA group (HEx400) with mast cell hyperplasia (arrows), **(D)** FAIC group (HEx400) showing erythrophagocytosis (arrow), and **(E)** FAICT group (HEx400) showing normal tissue. Comparison of **(F)** SOD activity in the spleen of the control, MI and treated mice, **(G)** CAT activity in the spleen of control, MI and treated mice, **(H)** MDA activity in the spleen of control, MI and treated mice, **(I)** Caspase-3 activity in the spleen of control, MI and treated mice. The results of biochemical assays are shown in mean ± SEM, *n* = 5 (“a” symbolizes *p* ≤ 0.05 normal control vs. experimental groups, “b” symbolizes *p* ≤ 0.05 Freund’s control vs. experimental groups, and “c” symbolizes *p* ≤ 0.05 MI vs. treatment group FAIC vs. FAICT).

The spleen of the control mouse showed normal architecture of red and white pulp (Figure 11A). The IP (Figure 11B) group showed erythrophagocytosis, while mast cell hyperplasia was observed in the FA group (Figure 11C). The erythrophagocytosis was also noted in the FAIC (Figure 11D) group. However, the FAICT group (Figure 11E) did not exhibit any pathological alteration.

##### 3.7.4.2. Biochemical changes in the spleen

SOD: SOD levels were lowered in the MI groups as compared to the control and the treatment groups. The decreased concentration of SOD was in the order of FA < IP < FAIC. The level of SOD was elevated in the FAICT group but was still lower than the control groups. The concentration of SOD in both the control groups was almost equal (Figure 11F; *p* ≤ 0.05).

CAT: The spleen showed very low levels of CAT in the MI groups when compared to the control groups. The lowering was in FA < IP < FAIC order. The Cin treatment exhibited increase in CAT level but the increase was not equipollent to the control groups. There was no significant difference between the FC and the NC groups (Figure 11G; *p* ≤ 0.05).

MDA: An increase in the LPO in the splenic tissues of the MI group was noted. The concentration of MDA was highest in the FA > IP > FAIC order. The treatment with Cin lowered LPO and brought it close to the control levels. The levels of MDA were same among the control groups (Figure 11H; *p* ≤ 0.05).

Cas 3: Cas 3 was found to be low in the splenic tissues of MI groups and was in FA > IP ≥ FAIC order. The Cin treatment increased Cas 3 but not equipollent to the control groups. The control groups did not exhibit any difference in Cas 3 (Figure 11I; *p* ≤ 0.05).

#### 3.7.5. Stomach

##### 3.7.5.1. Anatomical changes in the stomach

**Figure 12:**
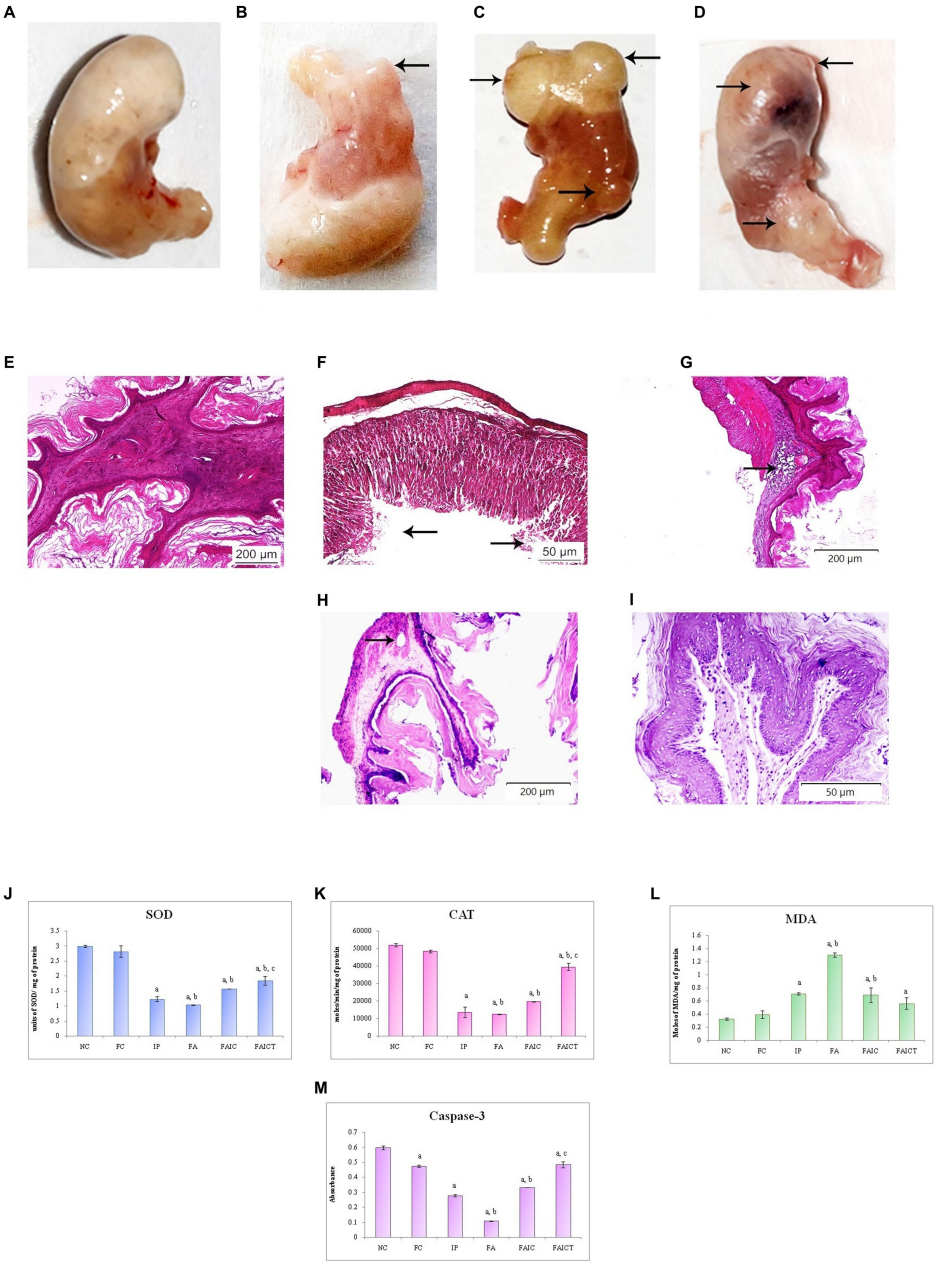
Stomach of the **(A)** control mouse, **(B)** IP group showing presence of the cyst/tumor as indicated by an arrow, **(C)** FA group showing the presence of multiple cysts/tumors (arrows), and **(D)** FAIC group with lesion and cysts/tumors (arrows) on the surface of cardiac stomach. T. S. stomach of the **(E)** control mouse (HEx100) showing normal histology, **(F)** IP group (HEx400) showing ulceration (arrows), **(G)** FA group (HEx40) showing a glandular cyst (arrow), **(H)** FAIC group (HEx40) showing the cyst (arrow), and **(I)** FAICT group (HEx200) showing normal histology. Comparison of **(J)** SOD activity in the stomach of the control, MI and treated mice, **(K)** CAT activity in the stomach of control, MI and treated mice, **(L)** MDA activity in the stomach of control, MI and treated mice, **(M)** Caspase-3 activity in the stomach of control, MI and treated mice. The results of biochemical assays are shown in mean ± SEM, *n* = 5 (“a” symbolizes *p* ≤ 0.05 normal control vs. experimental groups, “b” symbolizes *p* ≤ 0.05 Freund’s control vs. experimental groups, and “c” symbolizes *p* ≤ 0.05 MI vs. treatment group FAIC vs. FAICT).

The cyst/tumor on the stomach was observed in the IP group (Figure 12B) whereas in the FAIC group (Figure 12D), lesions at the antrum was noted. Unlike the control group (Figure 12A), the FA group (Figure 12C) showed the presence of the multiple cysts/tumors.

##### 3.7.5.2. Histopathological changes in the stomach

The normal arrangement of cells was seen in the stomach of control mice (Figure 12E), while IP group (Figure 1F) revealed ulceration in the surface epithelium. The presence of glandular cysts in the submucosal region of the cardiac stomach was observed in the FA (Figure 12G) group while a glandular cyst was noticed in the muscularis externa region of the FAIC group (Figure 12H). No histopathological changes were noticed in the stomach of FAICT group (Figure 12I).

##### 3.7.5.3. Biochemical changes in the stomach

SOD: The decreased SOD levels were recorded in the stomachs of the MI groups. Among the three MI groups, the concentration of SOD/mg of protein was the least in FA followed by IP and FAIC groups. Cin treatment elevated SOD level but could not revert it to normal (as control). The FC group showed no difference when compared to the NC group (Figure 12J; *p* ≤ 0.05).

CAT: The FA, IP and FAIC groups exhibited very low levels of CAT activity. The Cin treatment elevated the CAT level to some extent. The Freund’s control group did not show any difference in comparison to the normal control group (Figure 12K; *p* ≤ 0.05).

MDA: The highest MDA concentration was recorded in the FA group, followed by IP and FAIC. The treatment with Cin reduced the LPO in the FAICT group in comparison to the FA and IP groups. No difference in MDA enzyme activity was seen between the NC and FC (Figure 12L; *p* ≤ 0.05).

Cas 3: Cas 3 in gastric tissues was the lowest in the FA group, followed by the IP and FAIC groups. Cin treatment elevated the level of Cas 3 at par with the FC group. Furthermore, the FC group was found to have decreased Cas 3 activity than NC (Figure 12M; *p* ≤ 0.05).

#### 3.7.6. Heart

##### 3.7.6.1. Biochemical changes in the heart

**Figure 13:**
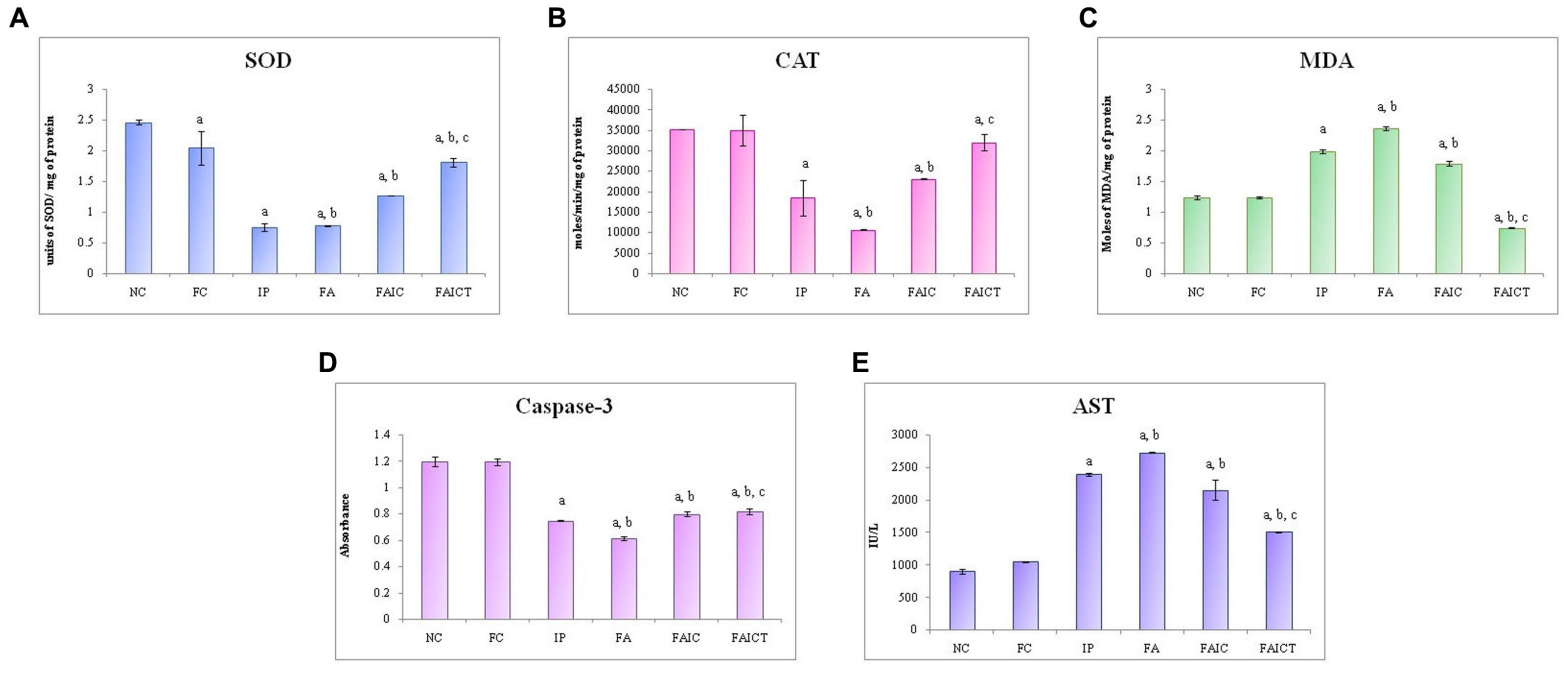
Comparison of **(A)** SOD activity in the heart of the control, MI and treated mice, **(B)** CAT activity in the heart of control, MI and treated mice, **(C)** MDA activity in the heart of control, MI and treated mice, **(D)** Caspase-3 activity in the heart of control, MI and treated mice, and **(E)** AST activity in the heart of control, MI and treated mice. The results of biochemical assays are shown in mean ± SEM, *n* = 5 (“a” symbolizes *p* ≤ 0.05 normal control vs. experimental groups, “b” symbolizes *p* ≤ 0.05 Freund’s control vs. experimental groups, and “c” symbolizes *p* ≤ 0.05 MI vs. treatment group FAIC vs. FAICT).

SOD: The cardiac tissues of the MI mice (IP, FA, and FAIC) had decreased SOD concentration in comparison to the treated and control mice. However, the level of SOD was almost equal in the IP and FA experimental groups and higher in the FAIC groups. The SOD concentrations increased in the treatment group (FAICT) when compared to the FAIC, IP and FA groups. The FC group showed a lowered SOD activity when compared to the NC (Figure 13A; *p* ≤ 0.05).

CAT: A decline in CAT activity was exhibited by tissues of the heart in all the MI groups. FA had the lowest CAT, followed by IP and FAIC groups. The Cin treatment brought CAT levels near to the FC. When compared to NC, FC did not exhibit any difference (Figure 13B; *p* ≤ 0.05).

MDA: The heart of the MI groups evidenced elevated levels of MDA in FA > IP > FAIC order. However, the MDA was extremely low (lower than control groups) in the Cin treated group. The FC group did not show any difference with respect to the NC group (Figure 13C; *p* ≤ 0.05).

Cas 3: A decrease in Cas 3 activity was noticed in the cardiac tissues of the MI groups in FA < IP < FAIC order. The treatment group (FAICT) showed an increase in Cas 3 activity when compared to the FA and IP groups, but the change was not significant with respect to the FAIC group. On the other hand, Cas 3 was lower in FAICT than the control groups. No difference was noted between the control groups (Figure 13D; *p* ≤ 0.05).

AST: An increase in AST was noted in the cardiac tissues of the MI groups (FA > IP > FAIC; Figure 13E) in comparison to the control groups, while AST decreased in the treatment group. No change was recorded in the FC group with reference to the NC group (*p* ≤ 0.05).

#### 3.7.7. Brain

##### 3.7.7.1. Histopathological changes in the brain

**Figure 14:**
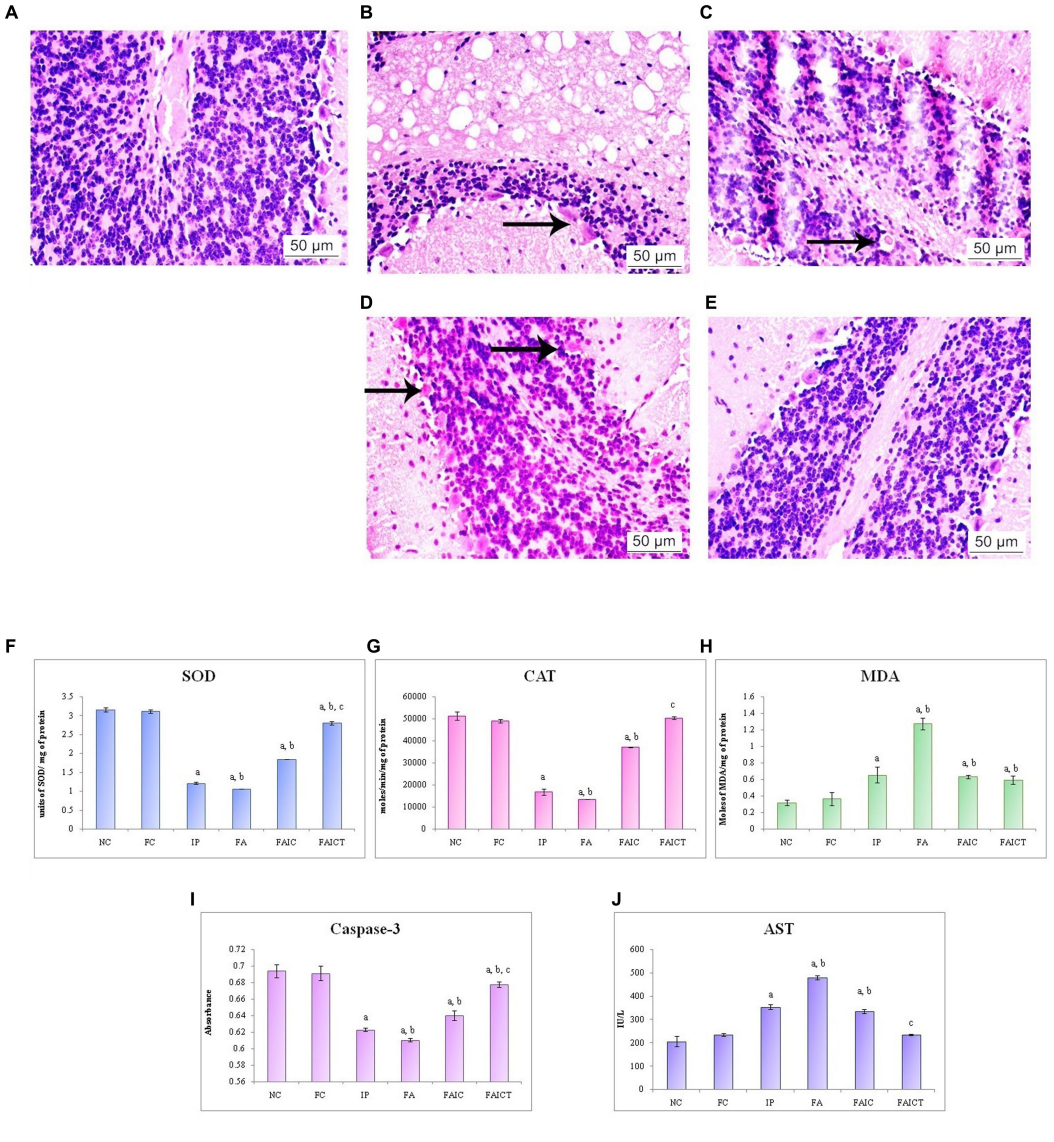
T. S. brain of the **(A)** control mouse (HEx400), **(B)** IP group (HEx400) showing axonopathy (indicated by an arrow), **(C)** FA group (HEx400) showing axonopathy (arrow), **(D)** FAIC group (HEx400) showing hemorrhage indicated by arrows, and **(E)** FAICT group (HEx400) showing no pathology. Comparison of **(F)** SOD activity in the brain of the control, MI and treated mice, **(G)** CAT activity in the brain of control, MI and treated mice, **(H)** MDA activity in the brain of control, MI and treated mice, **(I)** Caspase-3 activity in the brain of control, MI and treated mice, and **(J)** AST activity in the brain of control, MI and treated mice. The results of biochemical assays are shown in mean ± SEM, *n* = 5 (“a” symbolizes *p* ≤ 0.05 normal control vs. experimental groups, “b” symbolizes *p* ≤ 0.05 Freund’s control vs. experimental groups, and “c” symbolizes *p* ≤ 0.05 MI vs. treatment group FAIC vs. FAICT).

The brain evidenced various pathological conditions on administration of mycotoxin and co-administration of mycotoxin and adjuvant. Figure 14A shows the normal histology of the brain in a control mouse. The IP (Figure 14B) and FA (Figure 14C) groups displayed axonopathy. However, hemorrhage was recorded in the FAIC group (Figure 14D). No such pathologies were observed in Cin-treated mice (Figure 14E).

##### 3.7.7.2. Biochemical changes in the brain

SOD: SOD was lowered in all the three MI groups in brain tissues as well. Here, the reduction was more pronounced in FA and IP than in the FAIC group. The FAICT showed an increase in the SOD levels when compared to the MI groups but a decrease in comparison to the control groups. The FC group did not show any difference with respect to NC (Figure 14F; *p* ≤ 0.05).

CAT: CAT activity in the brain tissues of MI groups reduced in the order of FA < IP < FAIC. The Cin treatment brought the activity of CAT back to normal. FC did not exhibit any change when compared to NC (Figure 14G; *p* ≤ 0.05).

MDA: FA group showed maximum LPO in the tissues of the brain. The IP and FAIC group had lower LPO than FA. The FAICT group recorded a lower MDA level only when compared to FA and was equivalent to IP and FAIC. The FC group did not show any significant change in comparison to the NC group (Figure 14H; *p* ≤ 0.05).

Cas 3: Cas3 was found to be low in the FA, IP, and FAIC groups in the brain in FA > IP > FAIC order. Cas 3 was observed to be increased in the Cin treated group as compared to the MI groups. No significant difference was observed between the control groups (Figure 14I; *p* ≤ 0.05).

AST: The brain tissues of MI groups showed an increase in the AST (Figure 14J) concentration in FA > IP > FAIC order. The level of the enzyme, however, was lowered in the Cin treatment group (FAICT). The control groups (Freund’s and normal) did not have any notable differences (*p* ≤ 0.05).

#### 3.7.8. Testis

##### 3.7.8.1. Histopathological changes in the testis

**Figure 15:**
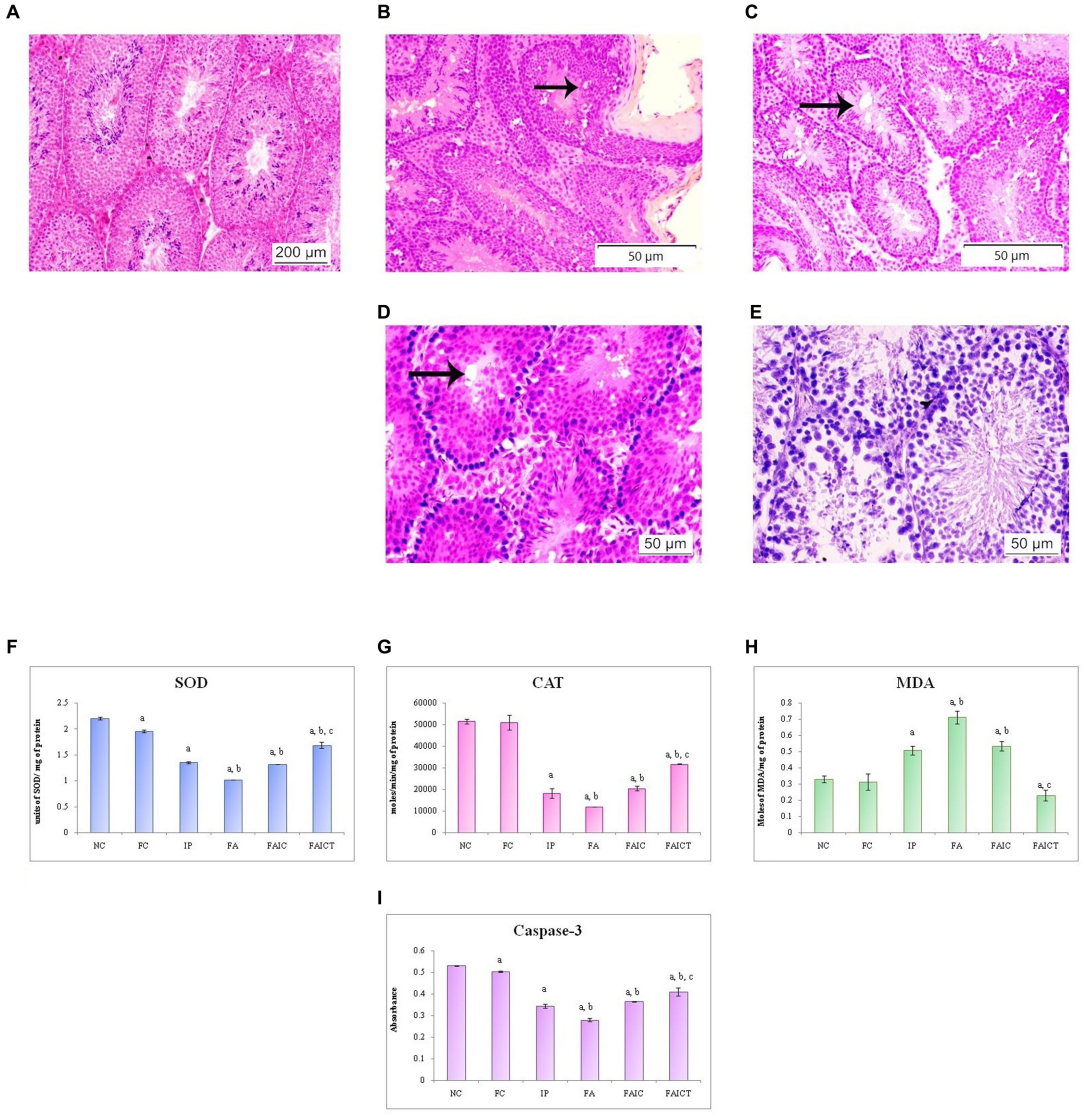
T. S. testis of the **(A)** control mouse (HEx100), **(B)** IP group (HEx200) showing rete testis hyperplasia (indicated by an arrow), **(C)** FA group (HEx200) showing vacuolation (indicated by an arrow), **(D)** FAIC group (HEx400) showing necrosis and vacuolation (arrow), **(E)** FAICT group (HEx400) showing normal cellular architecture. Comparison of **(F)** SOD activity in the testis of the control, MI and treated mice, **(G)** CAT activity in the testes of control, MI and treated mice, **(H)** MDA activity in the testes of control, MI and treated mice, **(I)** Caspase-3 activity in the testes of control, MI and treated mice. The results of biochemical assays are shown in mean ± SEM, *n* = 5 (“a” symbolizes *p* ≤ 0.05 normal control vs. experimental groups, “b” symbolizes *p* ≤ 0.05 Freund’s control vs. experimental groups, and “c” symbolizes *p* ≤ 0.05 MI vs. treatment group FAIC vs. FAICT).

Figure 15A shows the normal histology of the testis of a control mouse. The rete testis hyperplasia and vacuolation were observed in the testes of the IP (Figure 15B) and FA (Figure 15C) groups, respectively. The FAIC group, on the other hand, indicated the presence of necrosis as well as vacuolation (Figure 15D). The Cin-induced reparation was recorded in the treatment (FAICT) group (Figure 15E).

##### 3.7.8.2. Biochemical changes in the testis

SOD: The SOD level dropped in the testes of FA, IP, and FAIC groups. The FA had the lowest, whereas IP and FAIC had almost equal levels of SOD. The treatment group showed an increase in SOD when compared to the MI groups, but it was lower than the control group. On comparing both the control groups, SOD was found to be slightly lower in the Freund’s control group. No significant difference was noted in the control groups (Figure 15F; *p* ≤ 0.05).

CAT: The testes of MI groups showed a decrease in the CAT activity in FA > IP ≥ FAIC order. The Cin treatment (FAICT) increased the CAT activity to some extent, but it remained less than the control groups (NC and FC; Figure 15G; *p* ≤ 0.05).

MDA: MDA in the testis was found to be increased in all the three MI groups in the order of FA > FAIC ≥ IP. The FAICT exhibited a low (even lower than the control groups) concentration of MDA. The FC group showed no significant difference in comparison to NC (Figure 15H; *p* ≤ 0.05).

Cas 3: FA, IP, and FAIC groups showed a decrease in Cas 3 activity in comparison to the control groups (FA < IP < FAIC). Cin treatment (FAICT) increased Cas3 activity to some extent, but it was lower than the control (FC and NC) groups. The Freund’s control group showed lower Cas 3 than NC (Figure 15I; *p* ≤ 0.05).

## 4. Discussion

Cin, a bioactive component of cinnamon, gives it the ability to have therapeutic benefits against fungi, bacteria, diabetes, and other conditions (Ribeiro-Santos et al., 2017). In the present study, Cin at a concentration of 210 mg/kg BW/day through oral administration turned out to be a promising prophylactic agent against mycotoxicosis induced by TeA in combination with an adjuvant. TeA at 238 μg/kg BW was able to cause sub-chronic toxicity in mouse model. The criterion for selecting the dose for TeA and Cin was based on earlier reports by Kumari and Singh (2021). The range of dose for TeA and Cin was decided using IC_50_ values *in vitro* (unpublished data) and the determination of dose was decided using LD_50_ values *in vivo*.

Initially, the purpose of using Freund’s adjuvant in this study was to nullify the immunosuppressive effect of TeA. However, apart from being an immunostimulant; tissue damage and inflammation in the host animal have previously been associated with Freund’s adjuvants (Stils, 2005). Therefore, TeA when administered in combination with Freund’s adjuvant augmented the effect of TeA, resulting in more severe toxicity in the FA group.

The treatment with Cin was given to all the three MI groups: IP, FAIC, and FA. As the toxicity of TeA and the adjuvant was quite high in the case of the FA group, the treatment of Cin in the FA group did not turn out to be effective and, hence, has not been discussed here. The results for Cin prophylaxis in the IP group are part of another manuscript (Kumari and Singh, 2021), therefore, only one treatment group (FAICT) has been stated in this manuscript.

Kyphosis (hunchback) was noted in the FA group, which is a condition where the front-to-back curvature of the upper spine increases. Molds, mycotoxins, vitamin D deprivation, herd health status, and genetics have been evaluated as leading causes of kyphosis (Rortvedt and Crenshaw, 2012; DSM Lameness, 2022). However, specific reports on TeA toxicity leading to kyphosis have not yet been published.

Giambrone et al. (1978) documented reduced weight gain and poorer feed efficiency when TeA was administered to 3-week-old broilers through esophageal intubation. Decreased feed intake and increased body weight were noted in all the mycotoxicosis groups in the present study, with FA being the most affected group. Cin treatment on the other hand, reduced the toxic effect of TeA as evidenced in the present study. Earlier studies on the ameliorative effect of Cin have shown reduced body weight in obese male mice and rats that were fed a high-fat diet (Zuo et al., 2017; Rashwan et al., 2019).

Dysfunction of the liver leads to splenomegaly (Chapman et al., 2022). Increases in liver and spleen weights in this report may indicate hepatomegaly or splenomegaly (Kliegman, 2020; Chapman et al., 2022). Further, histopathological evidence of the liver confirms the presence of Non-alcoholic steatohepatitis (NASH), which explains the increased liver weight. Histology of spleen tissues also showed hyperplasia, which could be a reason for increase in spleen weight. The increased weight of the lungs in the MI groups was again supported by the histological studies which showed oedema and fibrosis. TeA induced increase in the weights of the kidney and stomach as well. Increased weight of the stomach was evident by the presence of tumors on the stomachs of MI groups, which was further confirmed by histological studies. The increased kidney weight could be attributed to the presence of crystals and inflammation in the renal tissues. The present study also documented an increased heart-to-body weight ratio. The reason for the increased weight of the heart could be associated with cardiomyopathy and other cardiac pathologies (Schoppen et al., 2020). Tumors, masses, and fluid build-ups in the brain may be associated with increased brain weight relative to body weight (Keep et al., 2012; Hoffman, 2020). However, no such results were recorded in the histological observations of the brain but axonopathy, hemorrhage, and increased oxidative stress were observed. Although TeA administration did not affect relative testis weight but a reduction in the relative weight was observed when TeA was given in combination with Freund’s adjuvant. The vacuolation and necrosis in histology also corroborated the same. Cin showed ameliorative effects in the present study and lowered the relative weights of the liver, lungs, kidneys, spleen, stomach, heart, and brain in comparison to the MI groups. Cin was able to reduce the toxicity of TeA in the lungs by reducing its relative weight. Similarly, bleomycin-induced elevated lung-to-body weight ratios were lowered when treated with Cin (Yan et al., 2018). A 7-week administration of Cin reduced heart weight/body weight and lung weight/body weight in cardiac hypertrophy induced by aortic banding (Yang et al., 2015). The neuroprotective role of Cin has also been reported in cerebral ischaemia (Zhao et al., 2015). In studies carried out by Wang et al. (2021), Cin has been indicated to attenuate liver and spleen weight indices in *Salmonella typhimurium* infection-induced injury in mice (Wang et al., 2021).

A significant decline in the granulocytes was noted in the Sprague–Dawley rats when fed with trichothecenes (Chattopadhyay et al., 2013). Differential leucocyte counts in this study exhibited neutropenia and lymphocytosis in the mycotoxicosis-induced groups as well. Similar results were reported by Kumari and Singh (2021) in which TeA mycotoxicosis resulted in elevated lymphocyte, monocyte, basophil and reduced neutrophil and eosinophil counts. The treatment with Cin maintained the percentage of agranulocytes and granulocytes close to the control groups. It was noteworthy that the increase in lymphocytes was more in the cases of IP and FAIC and not in FA, while all other results revealed more severe toxicity in the FA group. On the contrary, Figueiredo et al. (2022) noted no significant effect of Cin in the differential leucocyte count on the mice suffering from *Escherichia coli* induced-sepsis.

TeA in combination with alternariol, alternariol monomethyl ether, and tentoxin has been associated with hepatotoxic effects like necrosis and diseases such as cholestasis and phospholipidosis in HepaRG cells (Hessel-Pras et al., 2019). *In vivo* hepatotoxicity of TeA (with and without Freund’s adjuvant) was evident by hepatic steatosis and gross lesions in the MI groups in our study as well. Infiltration is the most commonly encountered inflammatory lesion of the liver in toxicity studies and is a form of chronic inflammation (Kumar and Pandey, 2018). In this study, TeA and TeA + adjuvant were found to cause foci of inflammatory cells in the liver of MI mice. In the present study, the hepatoprotective role of cinnamaldehyde was observed in the FAICT group as documented by Hussain et al. (2021) against acetaminophen-induced acute liver injury and Kumar and Pandey (2018) against isoniazid-induced hepatotoxicity in the mouse model.

Fumonisin B1 has been associated with porcine pulmonary oedema and pulmonary fibrosis in cases of chronic exposure (Pósa et al., 2013). Freund’s adjuvant-induced alveolar wall thickening might have decreased lung function and led to increased circumferential thickening of the airway wall (National Toxicology Program, 2014). In the present study, discoloration and gross lesions were observed in the lungs of MI groups due to TeA intoxication. The pulmonary oedema was noticed only when the TeA + Freund’s adjuvant was administered through the IP route. The intraluminal and mural bronchiolar fibroses in the lungs of the FAIC and IP groups were noted as well. Cin has reportedly been shown to have a potential anti-inflammatory impact in cases of acute lung damage (Huang and Wang, 2017). In the current study also, Cin preserved the histoarchitecture of the lung in the FAICT group, corroborating the same.

Nephrotoxicity has been reported in many species of rats and rabbits as the kidneys are the most sensitive target for mycotoxin exposure (Barnett and Cummings, 2018). The crystals often result from the administration of chemicals and/or metabolites that may precipitate in any segment of the nephron because of the concentration and pH of the urine filtrate (National Toxicology Program, 2014). In the present investigation, TeA intoxication induced cellular infiltration, formation of kidney crystals, and renal tubule inflammation in the kidneys of the IP, FA, and FAIC groups, respectively. Sharma et al. (2018) have reported the renoprotective role of Cin in food color-induced toxicity by reducing oxidative stress. Other natural compounds, such as oleic acid and ursolic acid, having nephroprotective effect were reported to counteract the nephrotoxicity induced by OTA (Zhang et al., 2021, 2023). Treatment with Cin restored the normal state of the renal tissues in our study as well.

Erythrophagocytosis usually takes place in order to phagocytize old or damaged erythrocytes by macrophages and is seen in the splenic red pulp (National Toxicology Program, 2014). Hemorrhage, red pulp hyperplasia, and apoptotic cells were observed in the spleen of chickens by Giambrone et al. (1978) when TeA was administered to young chickens via esophageal intubation. Similarly, TeA toxicity resulted in erythrophagocytosis in the IP and FAIC groups and hyperplasia (loose aggregates of well differentiated mast cells) in the FA group. Earlier reports by Datta et al. (2020) showed the defensive role of Cin against lead acetate induced-spleen toxicity in Swiss albino mice. No pathology was noted in the FAICT group, thus suggesting the protective effect of Cin in the spleen as well.

Earlier studies carried out on oral administration of TeA toxin showed cysts or tumor formation in the stomach (Kumari and Singh, 2021). The formation of cysts or tumors on the stomach was observed in this study as well, suggesting the tumorigenic property of TeA toxin. The compounds that cause ulcers are known to show toxicity through the flow of blood in the mucosa (National Toxicology Program, 2014). The detrimental effect of TeA ranges from hematological diseases to esophageal cancer (Meena and Samal, 2019). In the current study, TeA was found to cause gastric ulceration in the IP group, whereas the FA and FAIC groups showed the presence of the glandular cyst in the gastric tissues. The TeA induced-toxicity was significantly lower in the FAICT group. Cin again exhibited a gastroprotective role as evidenced in the histological and biochemical evaluations of the gastric tissues. The findings of the present study were supported by Tankam et al. (2013), who indicated that systematic intake of cinnamon powder could protect the stomach against non-steroidal anti-inflammatory drug-induced gastric ulcers through a cytoprotective mechanism.

Doi and Uetsuka (2011) reported that OTA resulted in acute depletion of striatal dopamine and its metabolites and neuronal cell apoptosis in the substantia nigra, striatum, and hippocampus. Concurrent with the studies of Doi and Uetsuka (2011), axonopathy and hemorrhage in the cerebellar region were documented in the present study due to TeA intoxication. Cin has earlier been reported to show neuroprotective effects in several models of brain disease. The neuroprotective role of Cin was explored against amyloid-β in neuronal SHSY5Y cell line (Emamghoreishi et al., 2019). The efficacy of Cin against TeA-induced pathological changes was seen in the brain as the FAICT group reinstated the normal architecture of brain cells.

Mycotoxins such as DON has been shown to lower the weights of seminal vesicle, epididymal and prostrate; reduce the spermatid count, testosterone concentration in serum; and induce morphological abnormalities in the sperm (Sprando et al., 2005). The co-treatment of ginger and cinnamon could reduce the CCl_4_-mediated damage in testicular tissues by enhancing its antioxidant capacity and lowering lipid peroxidation (Mazani et al., 2020). TeA-induced hyperplasia, vacuolation, and necrosis in the rete testis of mycotoxicosis groups. However, these pathologies were successfully repaired in the testis of Cin-treated group.

Oxidative stress in the various organs might be associated with a lack of coordinated upregulation of the antioxidant enzymes and discordance of their enzymatic activity (Omar, 2013). In mammalian tissues, the complex endogenous antioxidant system and chemical sequesters help to prevent oxidative damage. In our study, we explored the effect of the mycotoxin (TeA) on the activities of antioxidant enzymes in the tissues of liver, lungs, kidneys, spleen, stomach, heart, brain, and testis. The results showed that TeA administration altered the activities of antioxidant enzymes (SOD, CAT, and MDA). We further compared the co-administration of TeA with Freund’s adjuvant with two different dosing regimens. The induction of mycotoxicosis resulted in an elevated MDA level, indicating the presence of lipid peroxidation. Moreover, the decrease in CAT as well as SOD activities in the tissues further confirmed the occurrence of oxidative stress. The abnormal levels of AST and ALT suggested hepatocellular necrosis and leakage from the damaged tissues. Furthermore, the present study demonstrated the free radical scavenging activity of Cin by augmenting anti-oxidant enzymes and lowering lipid peroxidation, resulting in enhanced defense against TeA + Freund’s adjuvant-induced ROS (reactive oxygen species) production.

Cin has anti-proliferative activity against various types of human cancer cells (Li et al., 2016) and has been linked to both intrinsic (mitochondria-mediated) and extrinsic (death receptor–mediated) apoptoses (Lin et al., 2013). The increased levels of Cas-3 in the FAICT group also suggested the anti-proliferative role of Cin in this investigation. In mycotoxicosis-induced groups, the development of stomach tumors as well as splenic and testicular hyperplasia can be related to a decline in caspase 3 activity or a reduction in cell death. Similar to the present study, Zhao et al. (2017) reviewed that ochratoxin A (OTA)-induced nephrotoxicity was mediated via several pathways, including oxidative stress and apoptosis.

Therefore, this research advances our knowledge of how TeA + Freund’s adjuvant affects different organ responses. The severity of TeA toxicity was more when TeA + Freund’s adjuvant were injected into mice daily. However, the combined effect was mitigated when TeA + adjuvant was administered at 3-day intervals. Cin enhanced the antioxidant defense, protecting the mice against the apoptoses induced by TeA in combination with Freund’s adjuvant. Kyphosis, along with weight loss, decreased feed intake and an impaired antioxidant defense system in the FA group, implied that FA was the most affected mycotoxicosis group and TeA in combination with Freund’s adjuvant produced more severe toxic effects than TeA alone. Out of the eight organs investigated, the stomach, liver, and spleen had more pronounced histological alterations. However, confirmation of premalignant changes in the stomach, spleen and testes is desirous using immuno-histochemical studies and other tumor biomarkers. Although used as an immunopotentiator, the toxicity of Freund’s adjuvant has previously been reported. The present study for the first time reports the toxicity of Freund’s adjuvant in combination with TeA mycotoxin. Furthermore, Cin demonstrated a powerful inhibitory effect on TeA + Freund’s adjuvant-induced oxidative stress and had a protective effect on all eight organs; thus, can be employed as a preventive measure against TeA + Freund’s adjuvant-induced mycotoxicity.

## Data availability statement

The raw data supporting the conclusions of this article will be made available by the authors, without undue reservation.

## Ethics statement

The animal study was reviewed and approved by Institutional Animal Ethical Committee, Banaras Hindu University, India.

## Author contributions

All authors listed have made a substantial, direct, and intellectual contribution to the work and approved it for publication.

## Conflict of interest

The authors declare that the research was conducted in the absence of any commercial or financial relationships that could be construed as a potential conflict of interest.

## Publisher’s note

All claims expressed in this article are solely those of the authors and do not necessarily represent those of their affiliated organizations, or those of the publisher, the editors and the reviewers. Any product that may be evaluated in this article, or claim that may be made by its manufacturer, is not guaranteed or endorsed by the publisher.
