# Supplementary material for: Preventive role of cinnamaldehyde against tenuazonic acid- and Freund’s adjuvant-induced histopathological and biochemical alterations in the mouse model
Source: Front Microbiol. 2023 Jun 22;14:1159881. doi: 10.3389/fmicb.2023.1159881 (PMC10325827; doi:10.3389/fmicb.2023.1159881)
Supplement: Supplementary file 1 [file Data_Sheet_1.pdf]

Supplementary Material

Preventive role of cinnamaldehyde against tenuazonic acid- and Freund's adjuvant-induced histopathological and biochemical alterations in the mouse model

Ankita Kumari, Karuna Singh*

*** Correspondence:** Karuna Singh: karuna@bhu.ac.in

1 Supplementary Data

The extraction, isolation, and purification of tenuazonic acid from *Paradendryphiella arenariae* were performed using various chromatographic and spectroscopic studies. Thin-layer chromatography (TLC) and high-pressure liquid chromatography (HPLC) were performed for the isolation and characterization of the mycotoxin produced by *P. arenariae*. The structural elucidation was done using Fourier transform infrared (FT-IR) spectroscopy and nuclear magnetic resonance (NMR) studies. The quantitative determination of TeA was performed using HPLC and standard TeA. Further, the presence of TeA was confirmed through electron spray ionization mass spectrometry (ESI-MS) and high-resolution liquid chromatography-mass spectrometry (HRLCMS). The extraction and characterization data are part of another manuscript that has been submitted for publication.

2 Supplementary Figures

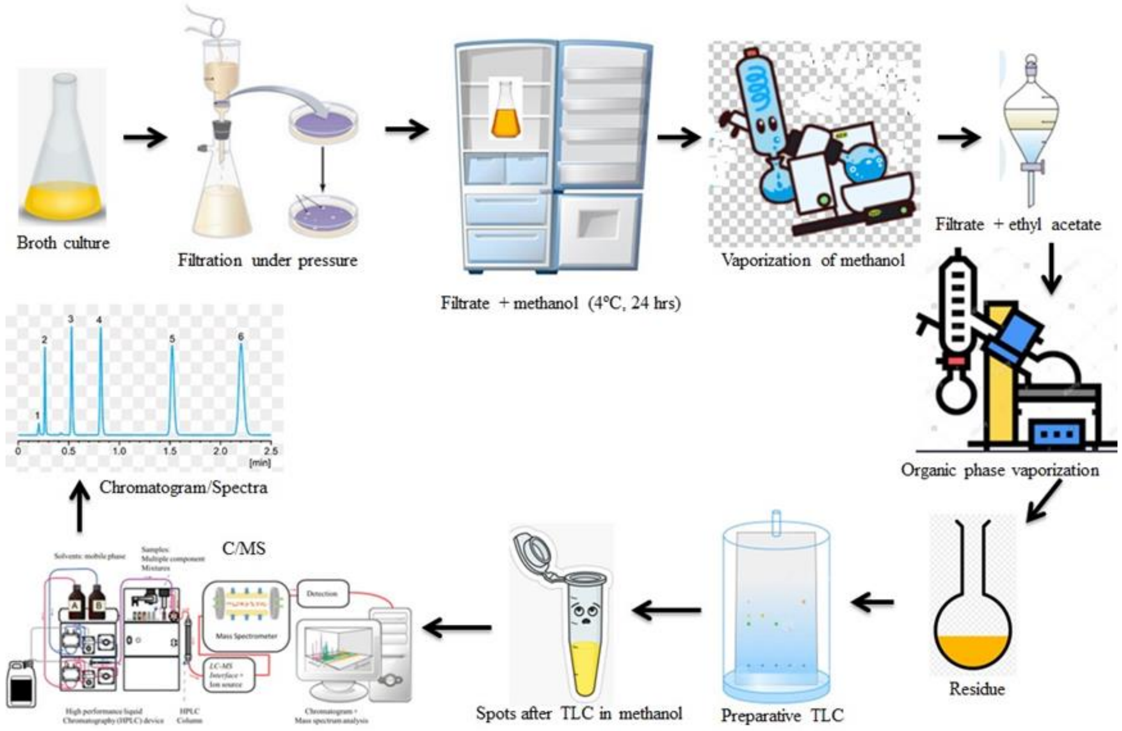

Supplementary Figure 1. Schematic representation for the extraction, isolation and purification of tenuazonic acid from *Paradendryphiella arenariae*.
